# Lewis Acids and Heteropoly Acids in the Synthesis of Organic Peroxides

**DOI:** 10.3390/ph15040472

**Published:** 2022-04-13

**Authors:** Ivan A. Yaremenko, Peter S. Radulov, Yulia Yu. Belyakova, Dmitriy I. Fomenkov, Svetlana B. Tsogoeva, Alexander O. Terent’ev

**Affiliations:** 1 N. D. Zelinsky Institute of Organic Chemistry, Russian Academy of Sciences, 47 Leninsky Prosp., 119991 Moscow, Russia; radulov.peter@mail.ru (P.S.R.); beljulka@inbox.ru (Y.Y.B.); cpl.fom@gmail.com (D.I.F.); 2 Organic Chemistry Chair I and Interdisciplinary Center for Molecular Materials (ICMM), Friedrich-Alexander University of Erlangen–Nürnberg, Nikolaus Fiebiger-Straße 10, 91058 Erlangen, Germany; svetlana.tsogoeva@fau.de

**Keywords:** Lewis acid, heteropoly acid, hydrogen peroxide, organic peroxides, catalysis

## Abstract

Organic peroxides are an important class of compounds for organic synthesis, pharmacological chemistry, materials science, and the polymer industry. Here, for the first time, we summarize the main achievements in the synthesis of organic peroxides by the action of Lewis acids and heteropoly acids. This review consists of three parts: (1) metal-based Lewis acids in the synthesis of organic peroxides; (2) the synthesis of organic peroxides promoted by non-metal-based Lewis acids; and (3) the application of heteropoly acids in the synthesis of organic peroxides. The information covered in this review will be useful for specialists in the field of organic synthesis, reactions and processes of oxygen-containing compounds, catalysis, pharmaceuticals, and materials engineering.

## 1. Introduction

Organic peroxides, due to their unique ability to form O-centered radicals via cleavage of the O-O bond, are widely used in polymer chemistry. In particular, dicumyl peroxide, dibenzoyl peroxide, 1,1-di-*tert*-butyl hydroperoxy cyclohexane, *tert*-butyl hydroperoxide, which are convenient in handling, have found application as initiators for low-temperature polymerization of styrene, butadiene, vinyl chloride, acrylates, ethylene [1,2], and as reagents for vulcanization of rubbers [3,4]. According to the latest research, the global organic peroxide market size was around US $2 billion in 2020 [5]. Despite the successful application of peroxides in the polymer industry, it was believed for a long time that the application of organic peroxides as drugs was not possible due to their low stability and the generation of hazardous reactive oxygen species, which can quickly and nonspecifically interact with biomolecules. Discovery of the natural peroxide Artemisinin (Qinghaosu) and its outstanding antimalarial activity [6,7] in 1972, showed that cyclic peroxides can be used in medicine as drugs. In 2015, Youyou Tu was awarded the Nobel Prize “for her discoveries regarding a new therapy for malaria” [8,9].

**Figure 1:**
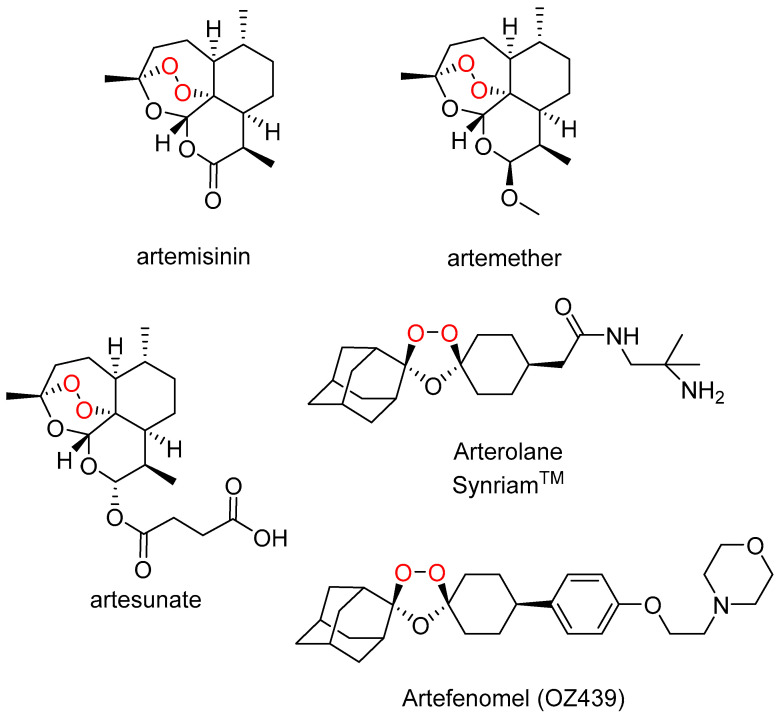
Artemisinin, its semi-synthetic derivatives, and synthetic Arterolane.

Drugs based on Artemisinin and its semisynthetic analogues are recommended by WHO as one of the most effective agents for the treatment of malaria (Figure 1) [10,11,12]. To overcome the emerging problem of drug resistance and to further improve the efficacy of Artemisinin, numerous derivatives of this unique natural product have recently been designed, synthesized and evaluated for biological activities [13,14].

The growing demand for Artemisinin has pushed scientists to develop its total synthesis. The disadvantage of the available methods for synthesis of Artemisinin is the low overall yield, which prompted the search for synthetic peroxides with antimalarial properties. Currently, the most promising classes of synthetic peroxides are 1,2-dioxolanes, 1,2,4-trioxolanes (ozonides), 1,2-dioxanes, 1,2,4-trioxanes, and 1,2,4,5-tetraoxanes. Representatives of these families have demonstrated antimalarial [10,11,15], anthelmintic [16,17,18,19,20,21,22,23,24,25,26,27,28], antitumor [29,30], anti-tuberculosis [31,32,33], growth regulatory [34,35,36] and fungicidal activity [37,38,39,40]. In 2012, arterolane (ozonide OZ277) was the first synthetic peroxide to be approved for treatment of malaria in medical practice (Figure 1) [41,42,43,44,45]. Ozonide artefenomel (OZ 439) is a second generation clinical candidate against malaria [46]. Very recently, it has been shown that arterolane exhibits in vitro activity against α-coronavirus NL63 and β-coronaviruses OC43, and SARS-CoV-2 [47,48]. Artemisinin and its derivatives were also found to be active against SARS-CoV-2 in vitro as well [49].

Modern approaches to the synthesis of organic peroxides are based upon the use of oxygen, ozone, and hydrogen peroxide as sources of the O-O group. The most common methods for the construction of the O-O group are the ene reaction of singlet oxygen with alkenes [50,51,52], [4 + 2], the cycloaddition of singlet oxygen to dienes [53,54], the peroxysilylation of alkenes by the Isayama-Mukayama reaction [55,56,57,58,59,60,61,62], the cyclization of unsaturated hydroperoxides by the Kobayashi reaction [63,64,65], processes with the participation of destabilized peroxycarbenium ions [66,67,68], the ozonolysis of alkenes [69,70,71,72,73,74], the nucleophilic addition of hydrogen peroxide to carbonyl compounds and their analogs catalyzed by acids [75,76,77,78,79,80,81,82,83,84,85,86,87,88,89,90,91], and the ring opening reaction of Donor–Acceptor cyclopropanes with alkyl hydroperoxides [92]. The most affordable starting materials for the synthesis of organic peroxides are carbonyl compounds and hydrogen peroxide.

**Figure 2:**
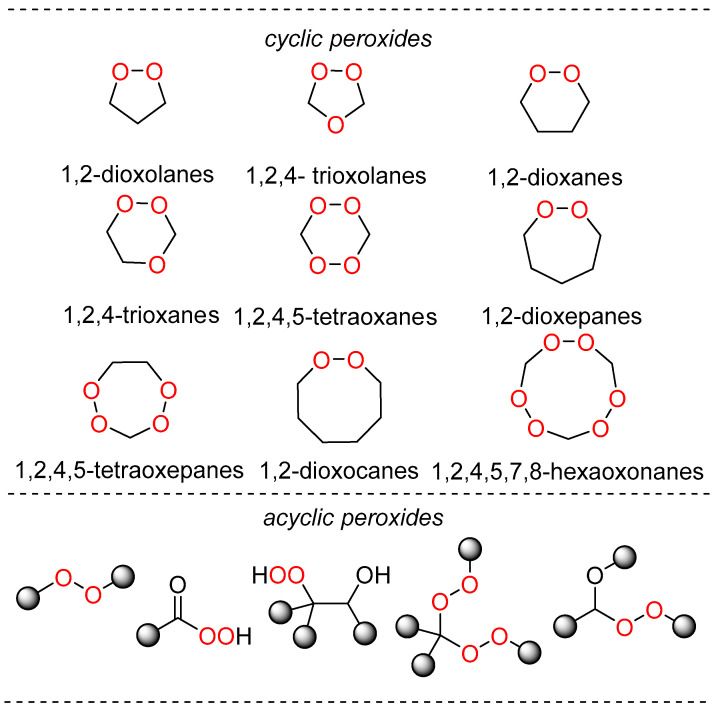
Reviewed cyclic and acyclic peroxides.

This review, which covers the major achievements in the synthesis of organic peroxides (Figure 2) using Lewis acids and heteropoly acids, consists of three parts: (1) metal-based Lewis acids in the synthesis of organic peroxides; (2) synthesis of organic peroxides promoted by non-metal-based Lewis acids; (3) application of heteropoly acids in the synthesis of organic peroxides. This review provides information that will be useful to specialists in the field of organic synthesis, catalysis, pharmaceuticals, and the polymer industry.

## 2. Metal-Based Lewis Acids as Catalysts in the Synthesis of Organic Peroxides

Traditionally, strong Bronsted acids play the role of a catalyst in the synthesis of organic peroxides. The use of metal-based Lewis acids for the synthesis of peroxides is a surprising phenomenon. Generally, peroxides decompose or rearrange under the action of transition metal salts [93,94]. However, some metal-based Lewis acids, on the contrary, promote the assembly of peroxides. In this section, we summarized the approaches on the synthesis of 1,2-dioxolanes, 1,2-dioxanes, 1,2-dioxepanes, 1,2-dioxocanes, 1,2,4,5-tetraoxanes, 1,2,4,5,7,8-hexaoxonanes, and acyclic peroxides under the action of metal-based Lewis acids.

### 2.1. Synthesis of Organic Peroxides Catalyzed by SnCl_4_, Me_2_SnCl_2_, SnCl_2_, and TiCl_4_

**Scheme 1:**
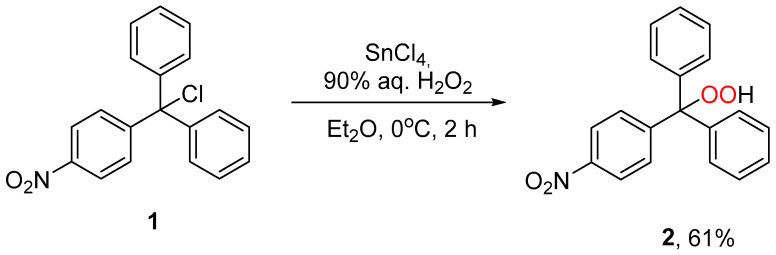
Synthesis of hydroperoxide **2**.

The first example of selective synthesis of organic peroxide using a metal-based Lewis acid SnCl_4_ as a catalyst goes back to 1950 [95]. Bartlett P. D. et al. carried out the nucleophilic substitution of the halogen atom in **1** with hydrogen peroxide in the presence of tin (IV) (Scheme 1). The hydroperoxide **2** formed granular crystals of a monohydrate melting at 99–101 °C with loss of water.

**Scheme 2:**
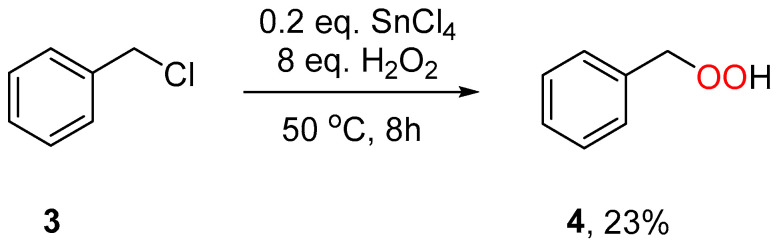
Synthesis of benzyl hydroperoxide **4**.

In 1959, R. Huttel et al. found that benzyl hydroperoxide **4** is formed by treating benzyl chloride **3** with an excess of hydrogen peroxide (90% aq. solution) in the presence of tin (IV) chloride as a catalyst [96]. The yield of benzyl hydroperoxide **4** was 23% (Scheme 2).

**Scheme 3:**
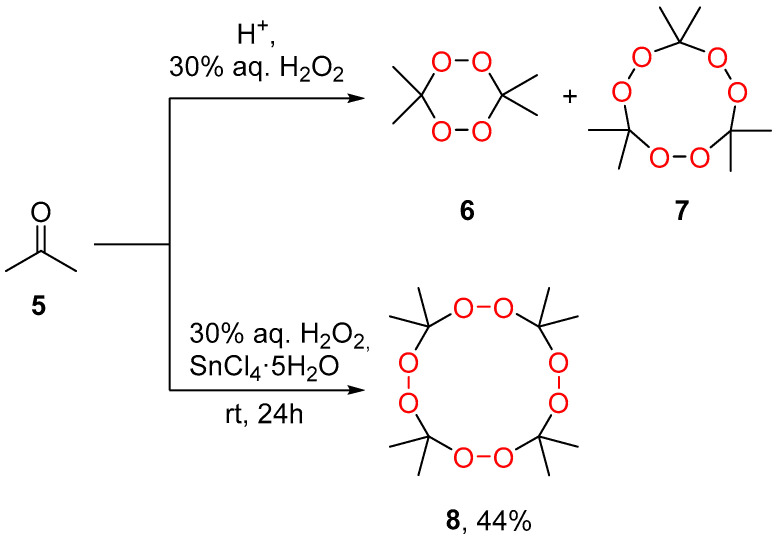
Synthesis of cyclic peroxides from acetone.

The reaction of acetone **5** with hydrogen peroxide in the presence of protic acid leads to the formation of tetraoxane **6** and hexaoxonane **7**. However, the peroxidation of acetone under the action of SnCl_4_ allows the obtaining of tetramer **8** in 44% yield (Scheme 3) [97]. Peroxide **8** was identified by molecular weight determination, elemental analysis, FTIR, NMR, and MS.

**Scheme 4:**
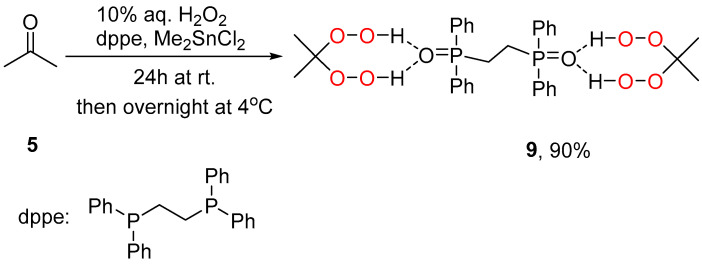
Synthesis of (bis(diphenylphosphinoyl)ethane)·2(2,2-dihydroperoxypropane) 1:2 adduct **9**.

The reaction of H_2_O_2_ with 1,2-bis(diphenylphosphino)ethane (dppe) in acetone in the presence of Me_2_SnCl_2_ leads to (bis(diphenylphosphinoyl)ethane)·2(2,2-dihydroperoxypropane) 1:2 adduct **9**, stabilized by hydrogen bonds between hydroperoxide groups and oxygen atoms of phosphorus groups (Scheme 4) [98].

**Scheme 5:**
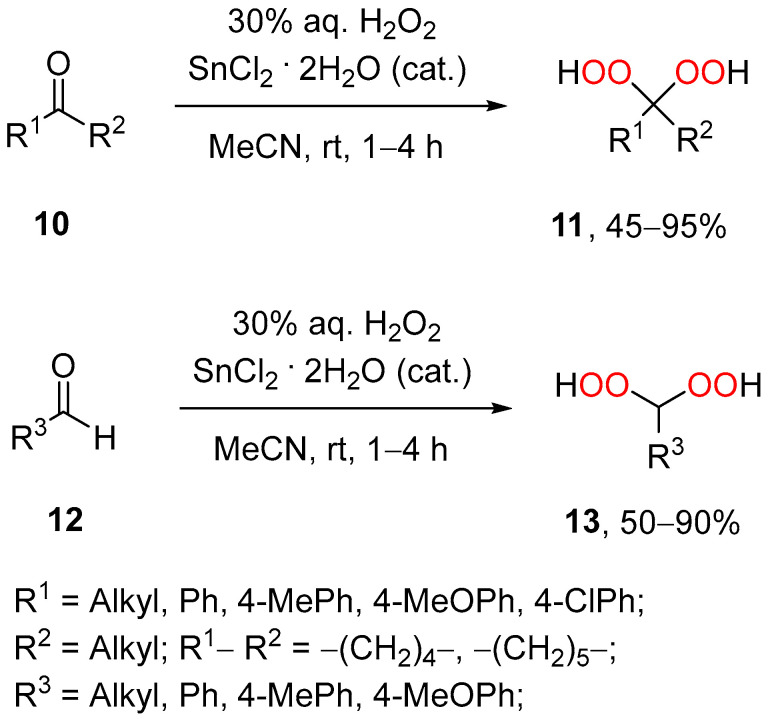
Synthesis of *gem*-bishydroperoxides **11**, **13** from ketones **10** and aldehydes **12**.

The synthesis of *gem*-bishydroperoxides **11** from ketones **10** was successfully carried out under the action of 30% aq. H_2_O_2_ using tin (II) chloride in catalytic amounts under mild conditions [91]. As starting substrates, cyclic or acyclic ketones, as well as substituted acetophenones and aldehydes, can be used. The yield of the peroxides **11** and **13** was 45–95% (Scheme 5).

**Scheme 6:**
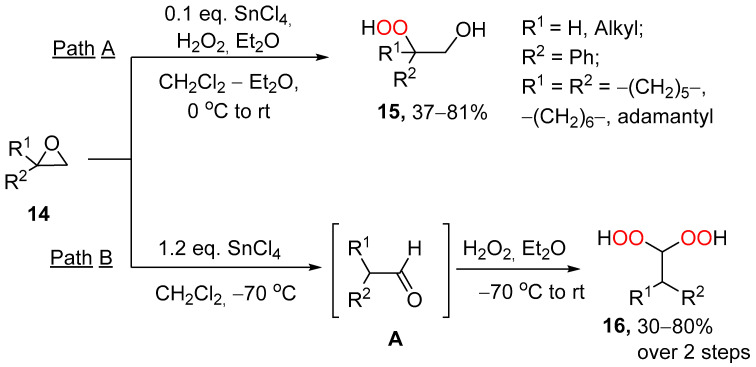
Transformation of substituted oxiranes **14** into the corresponding α-hydroxo-hydroperoxides **15** and geminal 1,1-dihydroperoxides **16**.

Substituted oxiranes **14** are easily transformed into the corresponding α-hydroxo-hydroperoxides **15** in good yield in the presence of a SnCl_4_-H_2_O_2_ system at 0 °C (Scheme 6, Path A) [99]. However, the opening of the oxirane ring at −78 °C, using SnCl_4_ in the amount of 1.2 eq., with the subsequent addition of an ethereal solution of H_2_O_2_ into the reaction, leads to the formation of geminal 1,1-dihydroperoxides **16**. This new pathway results from SnCl_4_-catalyzed rearrangement of oxiranes to aldehydes **A**, which then interact with hydrogen peroxide to form bishydroperoxides **16** (Scheme 6, Path B).

**Scheme 7:**
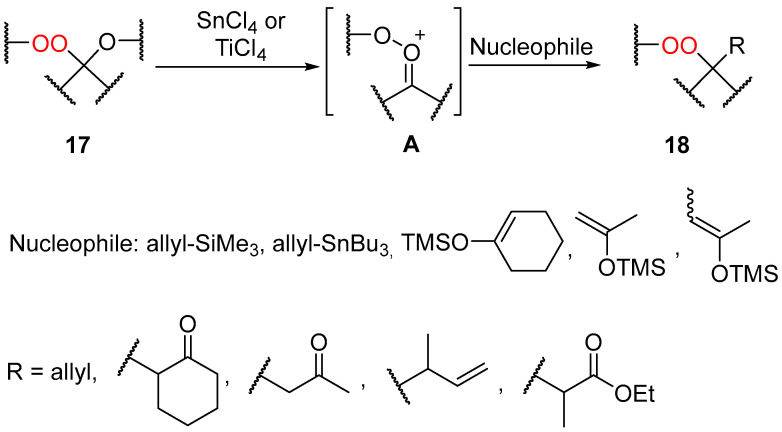
Synthesis of alkyl peroxides **18**.

Dussault P. et al. developed an approach to the synthesis of allylated peroxides, peroxyketones, and peroxyesters **18** by SnCl_4_ or TiCl_4_-mediated reaction of peroxyacetals **17** with electron-rich alkenes, proceeding via peroxycarbenium ion **A**. (Scheme 7) [100,101,102].

**Scheme 8:**
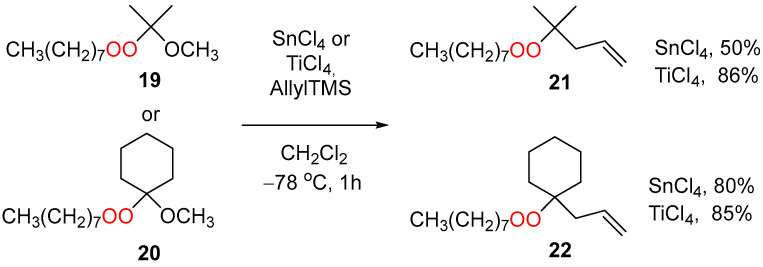
Allylation of peroxyacetals **19** and **20**.

Allylation of monoperoxyacetals **19**, **20** makes it possible to obtain peroxides **21**, **22** in good yield at −78 °C in methylene chloride. The reaction is catalyzed by TiCl_4_ and SnCl_4_ (Scheme 8) [100].

**Scheme 9:**
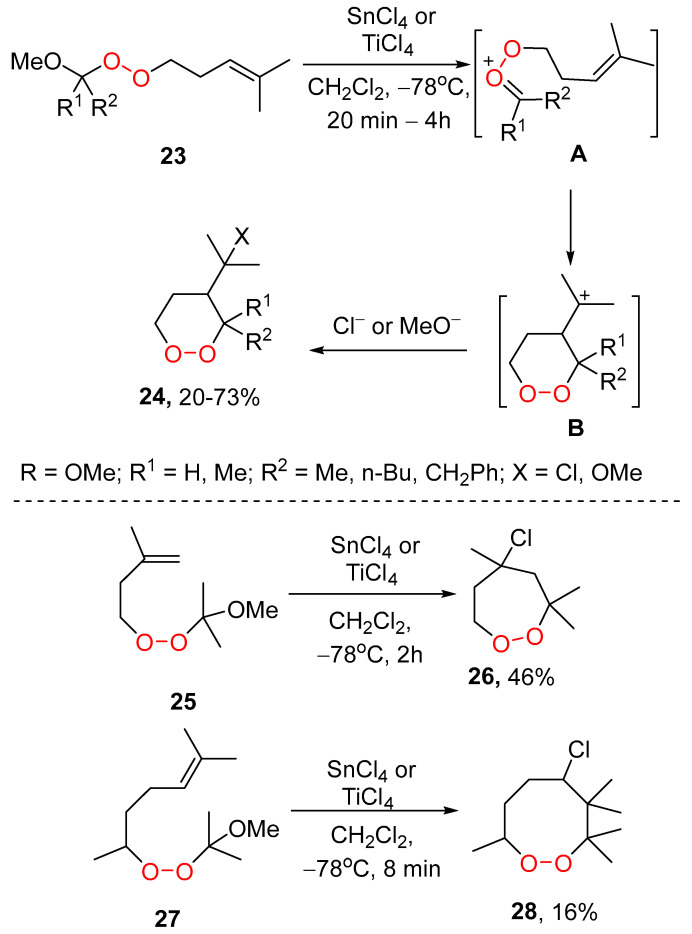
Intramolecular cyclization of peroxyacetals **23**, **25**, and **27**.

The transformation of alkyl peroxides under the action of Lewis acids has been described, where 1,2-dioxanes, 1,2-dioxepanes, and 1,2-dioxocanes are formed as target products [87]. Thus, intramolecular cyclization of peroxyacetals **23**, **25**, and **27**, containing an electron-rich double bond, occurs with the formation of cyclic peroxides **24**, **26**, and **28**, respectively, under action of 1 equiv. of TiCl_4_ or SnCl_4_ at −78 °C in CH_2_Cl_2_ at the N_2_ atmosphere (Scheme 9). The size of the peroxide ring depends on the position of the double bond in the starting alkyl peroxide.

**Scheme 10:**
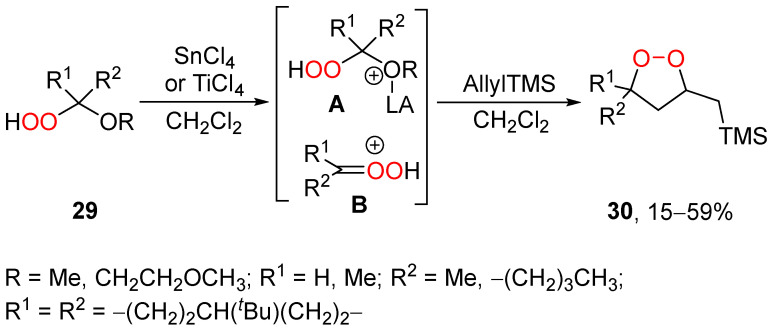
Synthesis of substituted 1,2-dioxolanes **30**.

The interaction of allyltrimethylsilane with α-alkoxyhydroperoxides **29**, promoted by SnCl_4_ and TiCl_4_, afforded with the formation of substituted 1,2-dioxolanes **30** (Scheme 10) [101].

**Scheme 11:**
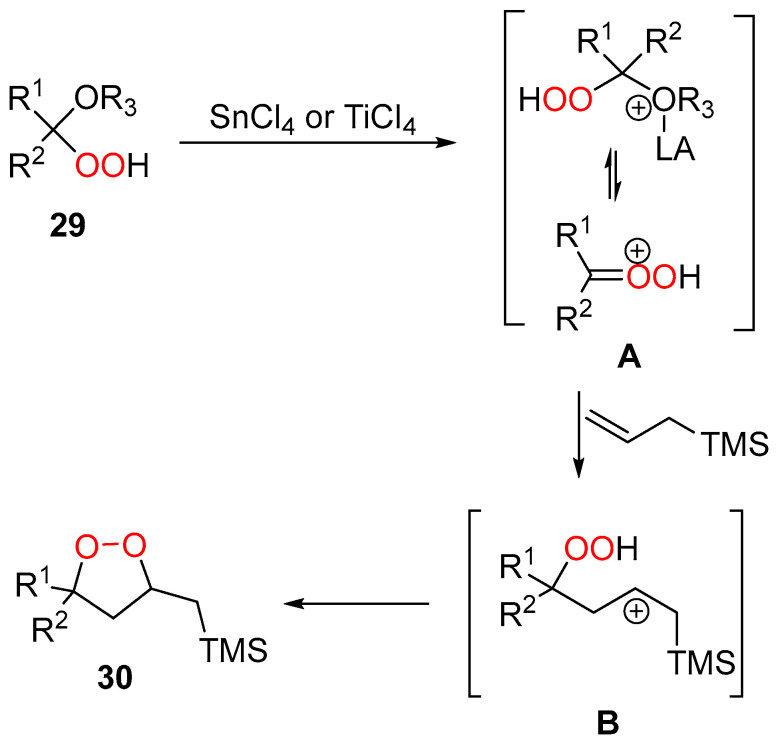
Probable mechanism of 1,2-dioxolane **30** formation.

The reaction mechanism of the formation of 1,2-dioxolane **30** includes the formation of hydroperoxycarbenium ion **A** peroxyacetal **29** under the action of SnCl_4_ or TiCl_4_ at the first step. Then hydroperoxycarbenium ion **A** undergoes nucleophilic attack by allyltrimethylsilane to form cation **B**, the cyclization of which leads to the 1,2-dioxolane **30** (Scheme 11) [101].

**Scheme 12:**
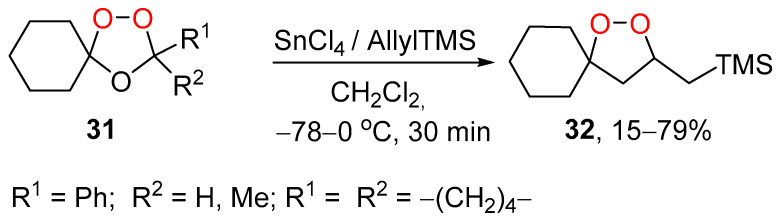
Synthesis of 1,2-dioxolanes **32** from ozonides **31**.

The above-mentioned approach was used to transform ozonides **31** into 1,2-dioxolanes **32**. This reaction proceeds under the action of SnCl_4_/AllylTMS system in a nitrogen atmosphere at the temperature range from −78 °C to 0 °C (Scheme 12) [103].

**Scheme 13:**
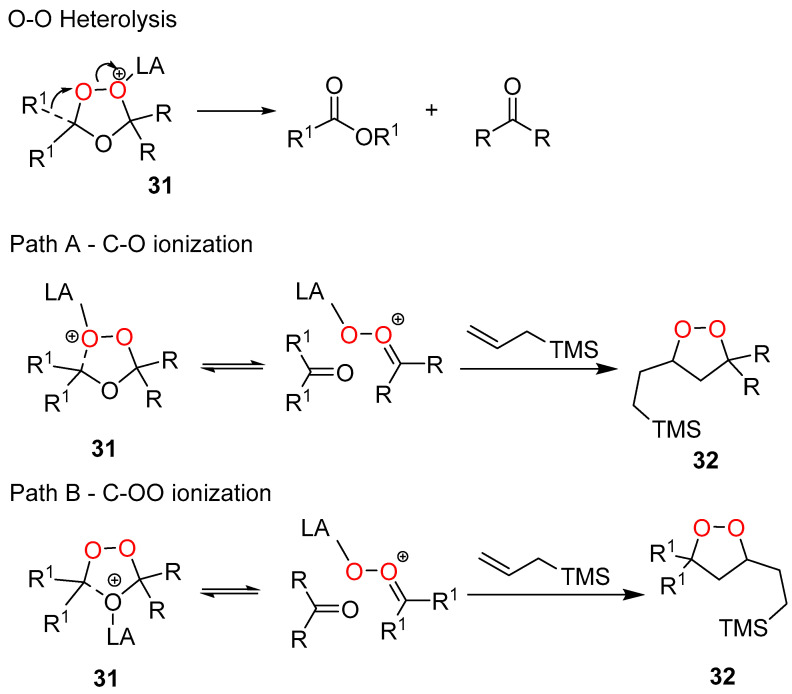
Mechanism of 1,2-dioxolanes formation **32** from ozonides **31**.

In the absence of allyltrimethylsilane, TiCl_4_ or SnCl_4_ can catalyze the heterolysis of the O-O-bond in ozonides. This reaction proceeds with the formation of the corresponding lactones and ketones (Scheme 13). The transformation of ozonides **31** into 1,2-dioxolanes **32** under the action of SnCl_4_ in the presence of allyltrimethylsilane proceeds through Path A and Path B, including the ionization of both C-O and C-OO bonds [104].

**Scheme 14:**
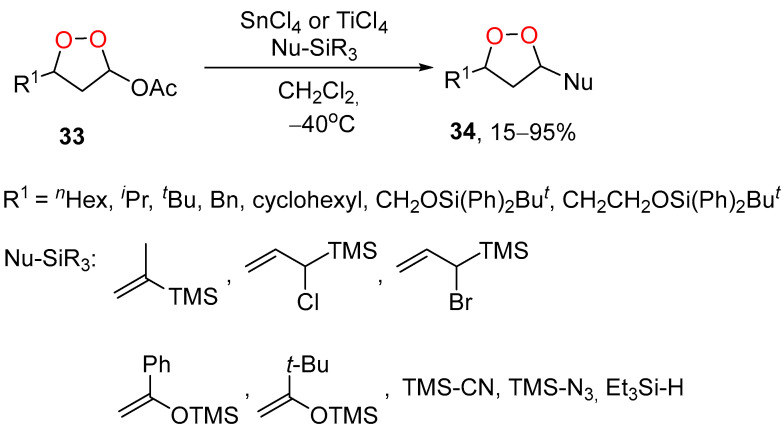
Synthesis of substituted 1,2-dioxolanes **34**.

The SnCl_4_ or TiCl_4_-mediated reaction between peroxyacetals **33** and electron-rich alkenes results in the formation of functionalized 3,5-disubstituted 1,2-dioxolanes **34** through the formation of a peroxycarbenium ion, which is attacked by the nucleophile. (Scheme 14) [105].

**Scheme 15:**
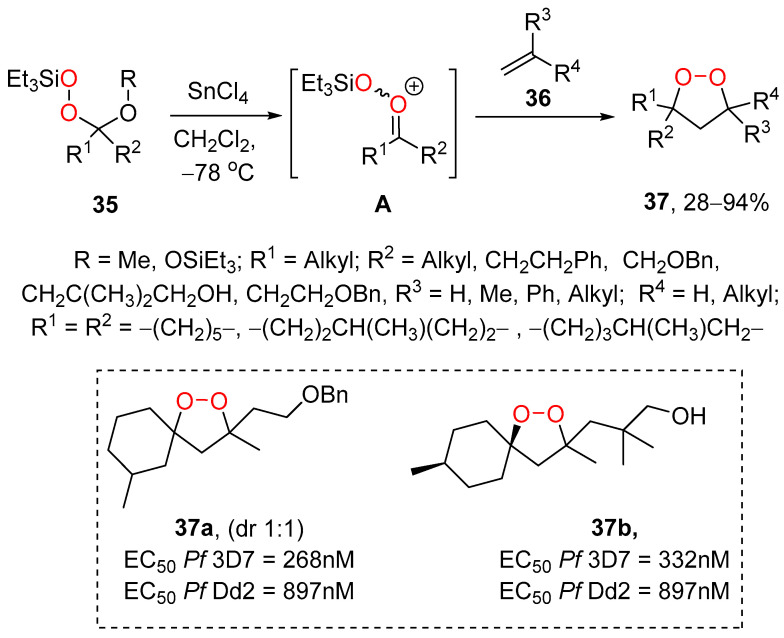
Synthesis and activity of 1,2-dioxolanes **37**.

The interaction of silylperoxyacetals **35** with alkenes **36** promoted by SnCl_4_ leads to in the formation of substituted 1,2-dioxolanes **37**. This process proceeds through the formation of the trimethylsilyl peroxycarbenium ion (Scheme 15) [82,106,107]. It was found that 1,2-dioxolanes **37a** and **37b** have a high antimalarial activity against *P. falciparum* [108].

**Scheme 16:**
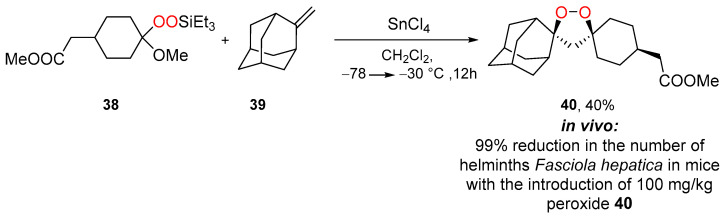
Synthesis and activity of 1,2-dioxolane (OZ78) **40**.

This approach was used for the synthesis of 1,2-dioxalane (OZ78) **40**, which exhibits high activity against *Fasciola hepatica* (Scheme 16) [109].

**Scheme 17:**
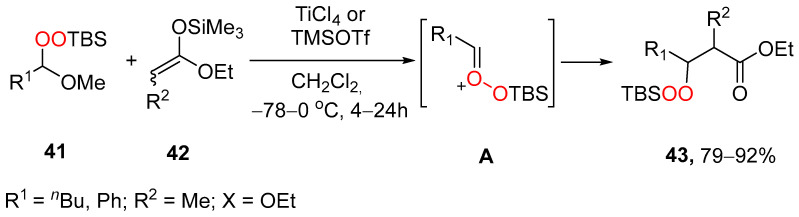
Synthesis of 3-peroxy-2-methylalkanoates **43**.

The reaction of peroxyacetal **41** with SKA (trimethylsilylketene acetal) **42** leads to the formation of peroxide **43**, containing ester functional group (Scheme 17) [102]. The best yield of peroxide **43** was achieved in the case of peroxyacetal **41** where R^1^ = Ph [110].

**Scheme 18:**
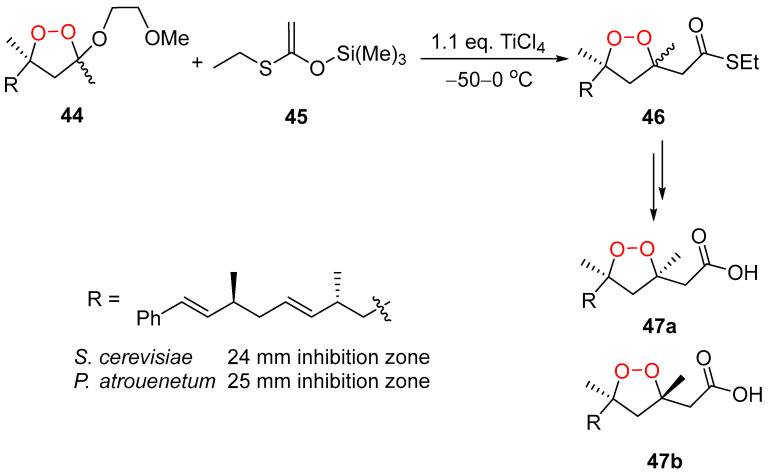
Synthesis of plakinic acid A **47a**,**b**.

Natural compounds with antitumor activity, such as stereoisomers of plakinic acids **47a,b**, were synthesized from peroxide **44** in three steps (Scheme 18). The key 1,2-dioxolane **46** in this sequence was synthesized from α-alkoxydioxolane **44** and O,S-ketene acetal **45** promoted by TiCl_4_ in 82% yield (Scheme 18). Both isomers of acid **47a,b** were isolated in individual form [111]. Also it was found that plakinic acids **47** inhibit the growth of the fungi *Saccharomyces cerevisiae* and *Penicillium atrounenetum* [39].

**Scheme 19:**
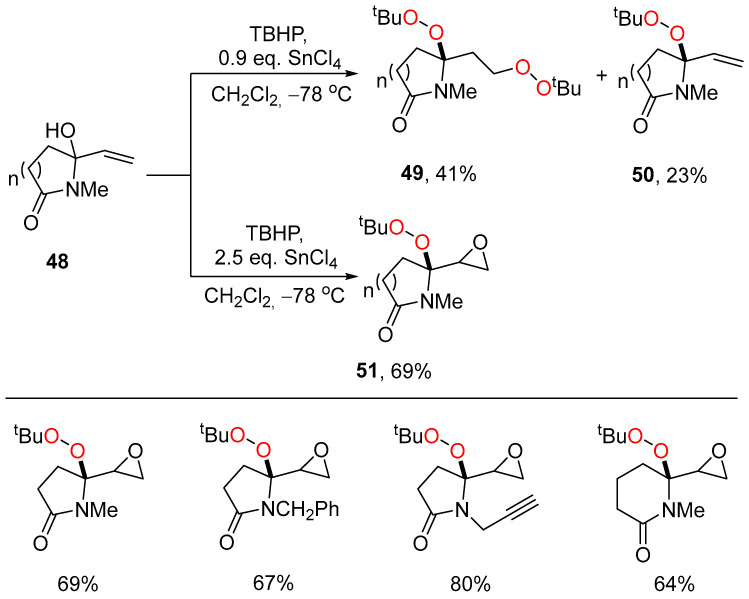
Synthesis of peroxides **49–51**.

It was found that the type of peroxidation product of allylic alcohols containing a lactam ring **48** depends on the amount of Lewis acid. Thus, peroxidation of alcohol **48** with the use of 0.9 eq. of SnCl_4_ leads to the formation of both mono- and diperoxides **49** and **50**. Increasing the amount of acid to 2.5 eq. with respect to **48** leads to the formation of epoxyalkyl peroxide **51** (Scheme 19) [112]. Anchimeric assistance of the hydroxyl group facilitates the addition of *tert*-butyl hydroperoxide to the double bond. Under the action of SnCl_4_, through the formation of a carbocation, the nucleophilic substitution of the hydroxyl group for *tert*-butyl peroxide occurs.

**Scheme 20:**
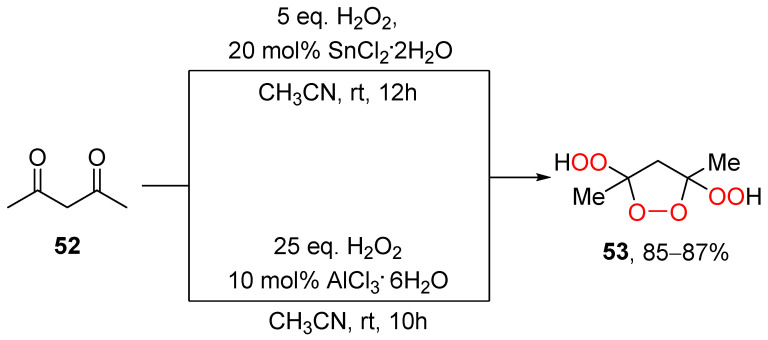
Synthesis of dihydroperoxy-1,2-dioxolane **53** from acetylacetone **52**.

Catalysis of the peroxidation reaction of acetylacetone **52** by strong protic acids (H_2_SO_4_, HClO_4_, HCl) leads to a complex mixture of cyclic and acyclic peroxides. However, with the use of SnCl_2_·2H_2_O and AlCl_3_·6H_2_O as a catalyst, the peroxidation of acetylacetone **52** proceeds selectively with the formation of dihydroperoxo-1,2-dioxolane **53** (Scheme 20) [113,114]. The reaction was carried out at a room temperature with 5–25 molar excess of 30% aq. solution of H_2_O_2_ and 10–20 mol% LA with respect to **52**.

**Scheme 21:**
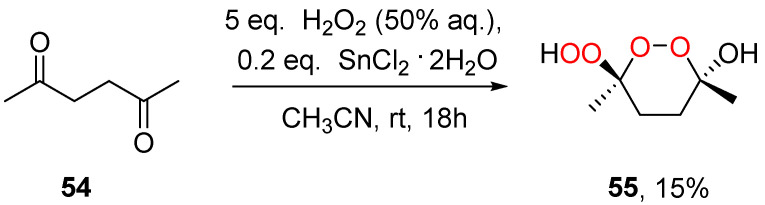
Synthesis of hydroxyhydroperoxy 1,2-dioxane **55** from 2,5-heptadione **54**.

The SnCl_2_·2H_2_O/H_2_O_2_ system was used in the peroxidation of 2,5-heptadione **54**. In this case, the reaction proceeds with the formation of hydroxyhydroperoxy 1,2-dioxane **55**, but in a low yield of target compound of 15% (Scheme 21) [115]. The reaction was carried out at a room temperature with 5-fold molar excess of 50% aq. solution of H_2_O_2_ and 20 mol. % SnCl_2_·2H_2_O with respect to **54**.

**Scheme 22:**
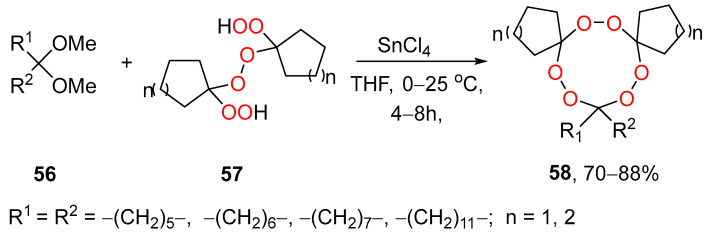
Synthesis of 1,2,4,5,7,8-hexaoxananes **58**.

The reaction of acetals **56** with 1,1′-dihydroperoxydi (cycloalkyl) peroxides **57** catalyzed by SnCl_4_ afforded with the formation of 1,2,4,5,7,8-hexaoxananes **58**. This approach makes it possible to solve the problem of the synthesis of hexaoxonanes from cycloalkanones with ring sizes C6–C8 and C12 (Scheme 22) [88].

### 2.2. Peroxidation of Ketones and Aldehydes in the Presence of MeReO_3_

**Scheme 23:**
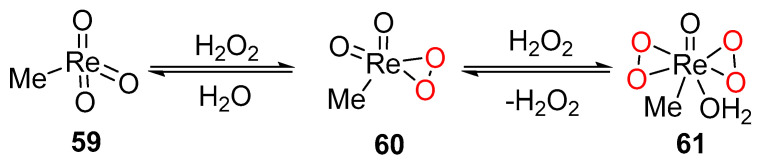
Formation of the peroxo complexes MeReO_3_.

The proposed mechanism of ketone peroxidation by the H_2_O_2_/MeReO_3_ system is based on the coordination of hydrogen peroxide with rhenium, which acts as a Lewis acid with the formation of peroxocomplex **61** (Scheme 23) [116,117,118]. The resulting peroxocomplex **61** interacts with a carbonyl compound with the transfer of a peroxo group. Furthermore, MeReO_3_ can react with a carbonyl group, as a Lewis acid to activate the carbonyl carbon atom.

**Scheme 24:**
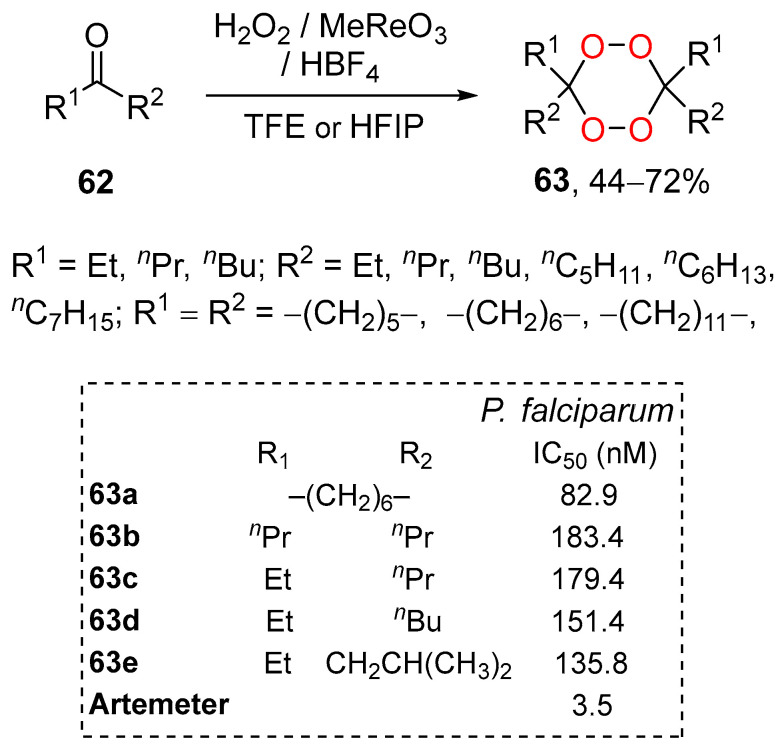
Synthesis and antimalarial activity of 1,2,4,5-tetraoxanes **63**.

The addition of HBF_4_ to the 30% aq. H_2_O_2_/MeReO_3_ system leads to the formation of symmetric 1,2,4,5-tetraoxanes **63** from cyclic ketones **62**, as well as 3,3,6,6-tetraalkyl-1,2,4,5-tetraoxanes **63** from unsymmetrical ketones **62**, respectively (Scheme 24) [119]. 1,2,4,5-Tetraoxanes **63a–e** exhibit antimalarial activity against the chloroquine-resistant strain of *P. falciparum*.

**Scheme 25:**
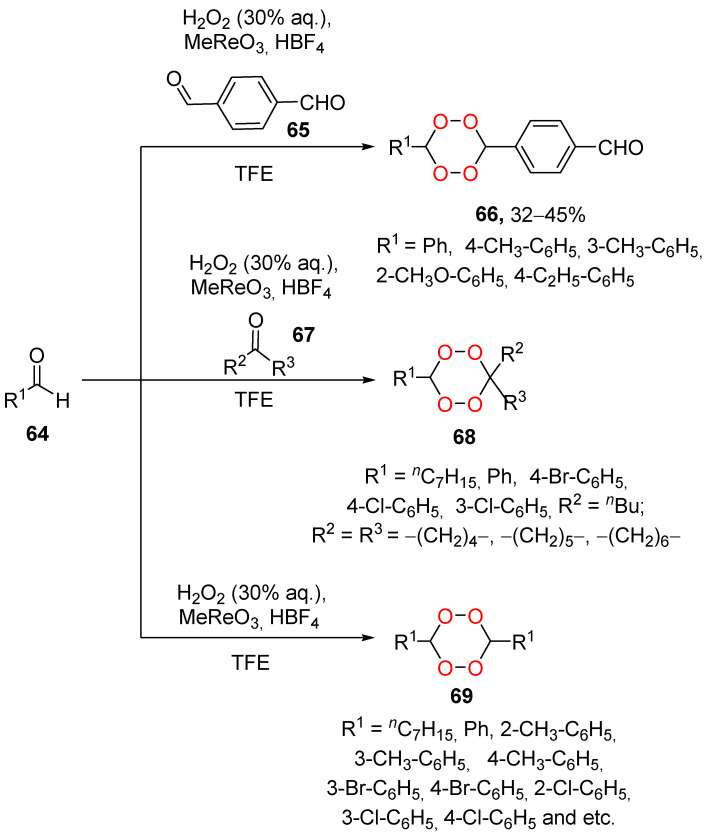
Synthesis of tetraoxanes **66, 68** and **69** from aldehydes **64**.

The combination 30% aq. H_2_O_2_/MeReO_3_/HBF_4_ in TFE is an effective system for the synthesis of both symmetric **69** and non-symmetric tetraoxanes **66** or **68** from aldehydes **64** in good yields (Scheme 25) [120,121,122]. It was found that such tetraoxanes exhibit antimalarial activity in vitro.

**Scheme 26:**
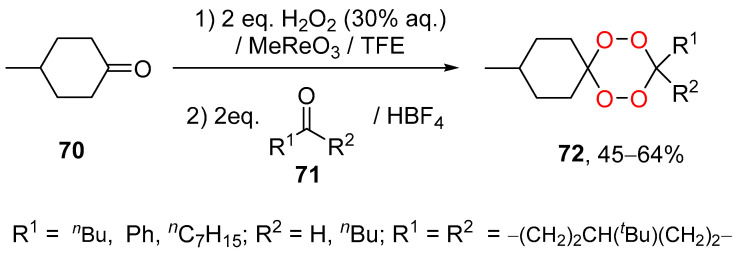
Synthesis of unsymmetrical 1,2,4,5-tetraoxanes **72**.

Non-symmetric tetraoxanes **72** were synthesized from 4-methyl cyclohexanone **70** and ketone or aldehyde **71** under the action of H_2_O_2_ in the presence of 1 eq. of HBF_4_ and 0.1 mol% of MeReO_3_ with respect to the starting ketone in TFE medium. (Scheme 26) [122].

**Scheme 27:**
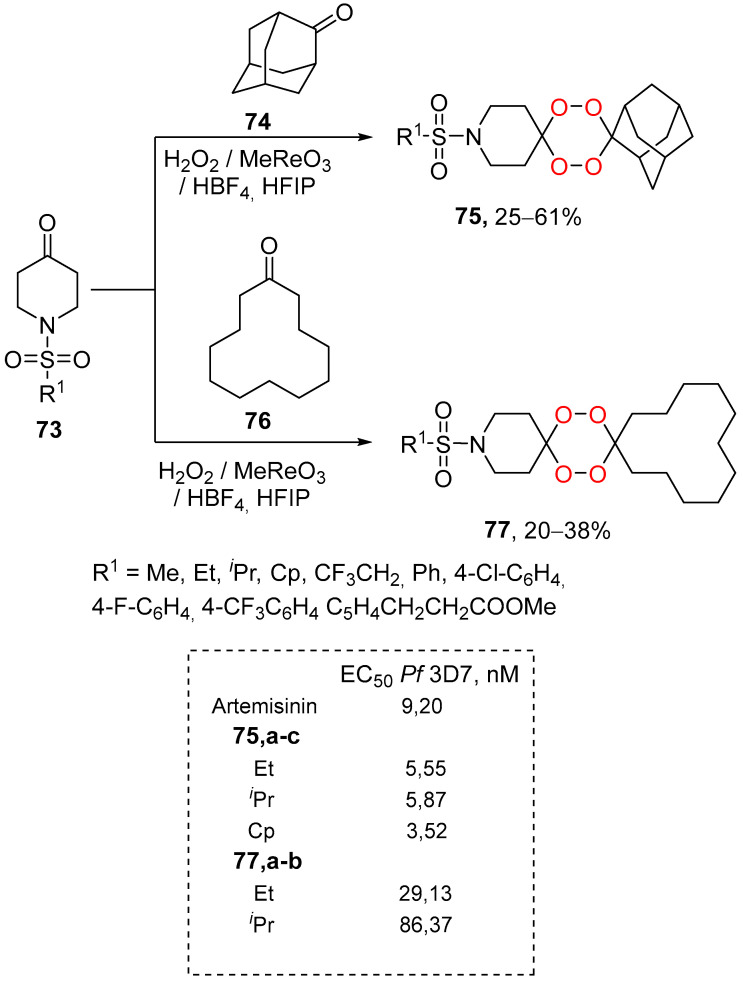
Synthesis of non-symmetric 1,2,4,5-tetraoxanes **75** and **77**.

The interaction of sulfonylpiperide-4ones **73** with ketones **74**, **76** promoted by H_2_O_2_/MeReO_3_/HBF_4_ in HFIP leads to the formation of non-symmetric 1,2,4,5-tetraoxanes **75** and **77**, which exhibit high antimalarial activity (Scheme 27) [123].

### 2.3. Sc(OTf)_3_, Yb(OTf)_3_, InCl_3_ and In(OTf)_3_ in the Synthesis of Organic Peroxides

**Scheme 28:**
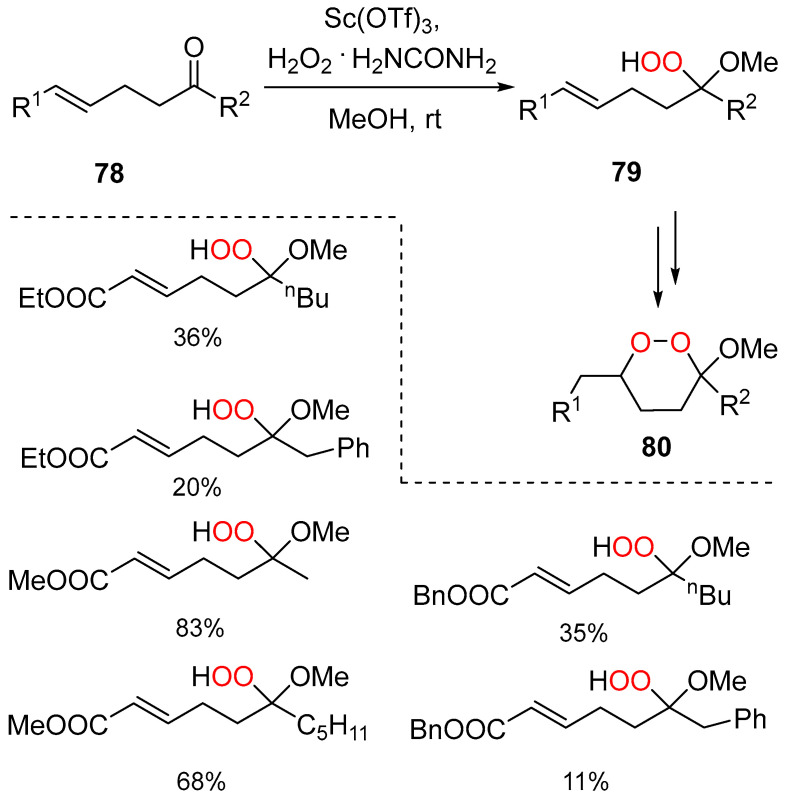
Synthesis of alkoxyhydroperoxides **79**.

In 2001, Kobayashi and colleagues developed a new method for the synthesis of alkoxyhydroperoxides **79** based on the reaction of the carbonyl group of unsaturated ketones **78** with H_2_O_2_·H_2_NCONH_2_, catalyzed by Sc(OTf)_3_. Cyclization of alkyl hydroperoxides **79** leads to 1,2-dioxanes **80** according to the Michael reaction. This method allows for the obtaining of substituted cyclic peroxides containing various functional groups in their structure (Scheme 28) [63,64,65,124].

**Scheme 29:**
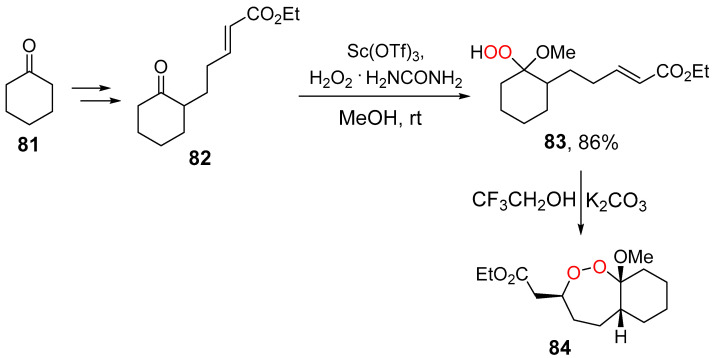
Synthesis of cyclic peroxide **84**.

The system H_2_O_2_·H_2_NCONH_2_/Sc(OTf)_3_ was used in the synthesis of peroxyacetal **83**, which under basic conditions undergoes intramolecular cyclization with the formation of cyclic peroxide **84** (Scheme 29) [125].

**Scheme 30:**
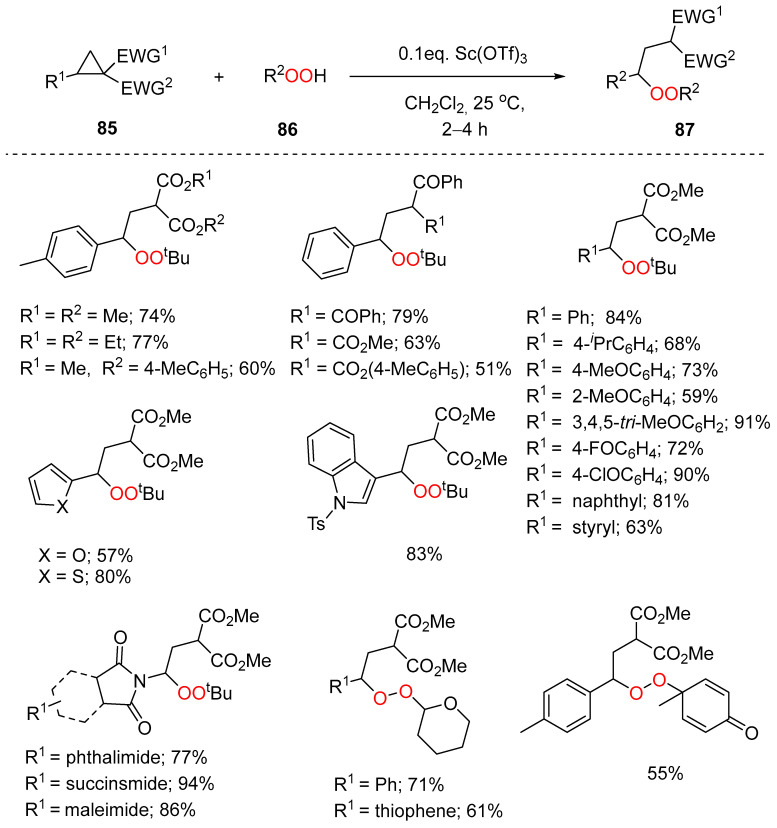
Synthesis of peroxides **87**.

Recently, Saha et al. found that the ring opening of Donor–Acceptor (D–A) cyclopropanes **85** in the presence of *^t^*BuOOH or hydroperoxides **86** under the action of Sc(OTf)_3_ leads to the formation of various peroxides **87** in 51–91% yield (Scheme 30) [92]. The reaction can be carried out on a gram scale in 74% yield of the target peroxide.

**Scheme 31:**
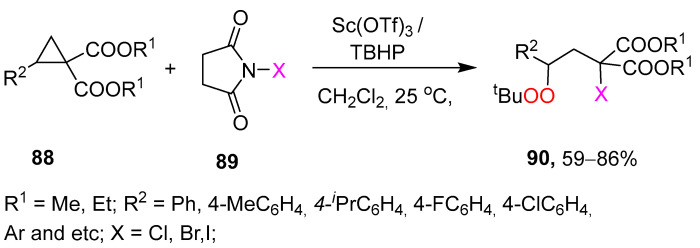
Synthesis of haloperoxides **90**.

The interaction of Donor–Acceptor cyclopropane **88** with *^t^*BuOOH and *N*-halosuccinimides **89**, which acts as a source of halogen, provides haloperoxides **90** in moderate to good yields (Scheme 31) [92].

**Scheme 32:**
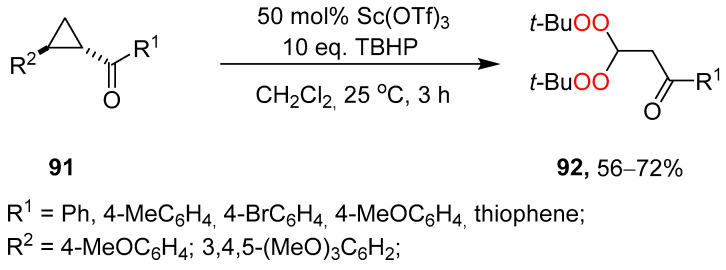
Synthesis of bis-*tert*-butyl peroxides **92**.

It is noteworthy that the interaction of cyclopropanes **91** containing one acceptor substituent with *tert*-butyl hydroperoxide under the action of 0.5 eq. Sc(OTf)_3_ leads to bis-*tert*-butyl peroxides **92** in 56–72% yields (Scheme 32) [92].

**Scheme 33:**
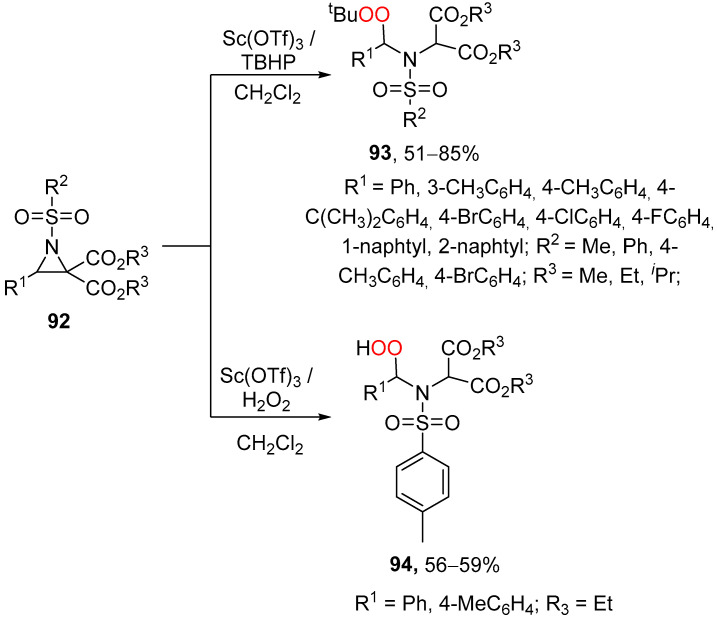
Synthesis of peroxides **93** and **94** from donor-acceptor aziridines **92**.

The use of the H_2_O_2_ or TBHP/Sc(OTf)_3_ system for ring opening of donor-acceptor aziridines **92** leads to α-sulfanilamido peroxides **93** and **94** in good yield (Scheme 33) [126]. The reaction can be scaled up to the grams in 70% yield.

**Scheme 34:**
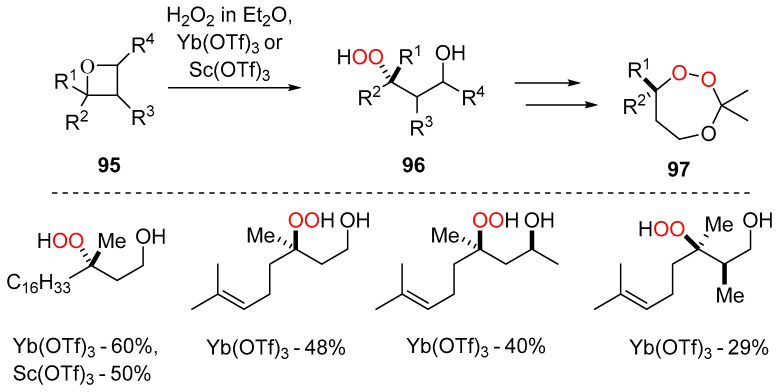
Synthesis of hydroperoxides **96** from oxetanes **95**.

In 2002, Dussault et al. [127] demonstrated the ring opening of oxetanes **95** with an ethereal solution of H_2_O_2_, catalyzed by Yb(OTf)_3_ and Sc(OTf)_3_ with the formation of peroxides **96**, which act as intermediates in the synthesis 1,2,4-trioxepanes **97** (Scheme 34).

**Scheme 35:**
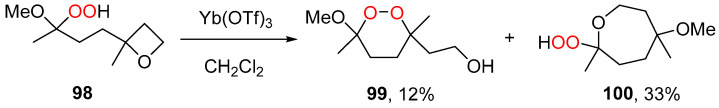
Synthesis of peroxides **99** and **100** from hydroperoxyoxetane **98**.

Hydroperoxyoxetane **98** rearranged into endoperoxide **99** in 12% yield and exoperoxide **100** in 33% yield under the action of Yb(OTf)_3_ in methylene chloride (Scheme 35) [74].

**Scheme 36:**
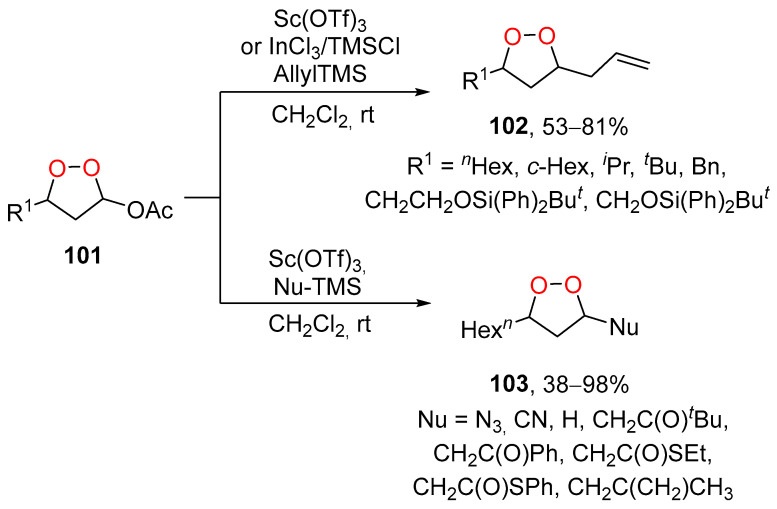
Synthesis of 1,2-dioxolanes **102** and **103** from peroxyacetals **101**.

The use of catalytic amounts of Sc(OTf)_3_ or InCl_3_ in the reaction of endoperoxyacetals **101** with allyltrimethylsilane (AllylTMS) and its derivatives (Nu-TMS) makes it possible to obtain 3,5-disubstituted-1,2-dioxolanes **102** and **103** by the Sakurai reaction. Sc(OTf)_3_ or InCl_3_ allow the reaction to be carried out under milder conditions than when using SnCl_4_ and TiCl_4_ (Scheme 36) [128,129].

**Scheme 37:**
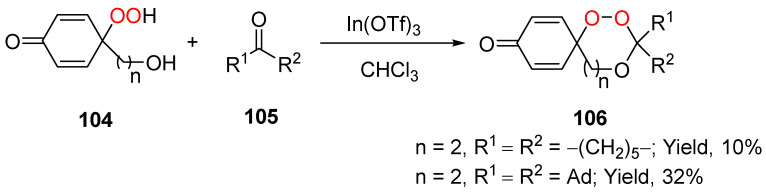
Synthesis of 1,2,4-trioxepanes **106**.

Cyclic peroxides such as spiro 1,2,4-trioxepanes **106** were obtained from hydroperoxides **104** and ketones **105** by using Indium (III) triflate as a catalyst (Scheme 37) [130].

### 2.4. Mercury Salts in the Synthesis of Peroxides

**Scheme 38:**
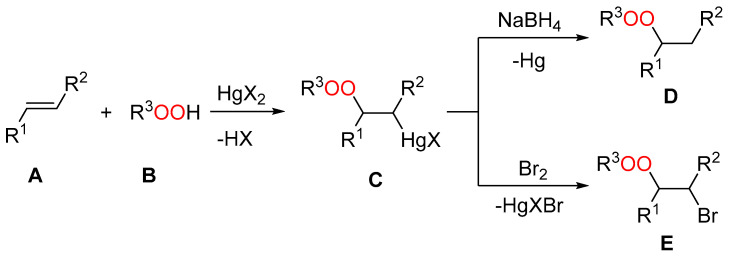
Synthesis of acyclic peroxides **D** and **E**.

In a process known as peroxymercuration, alkyl peroxides **D**, **E** can be prepared from alkenes **A** and alkyl hydroperoxide **B** in the presence of a suitable mercury (II) salt (Scheme 38). In this case, mercury salts act as a mild electrophilic reagent. The interaction of mercury (II) salt with an alkene leads to cationic species, which reacts with alkyl hydroperoxide to form mercurylalkyl peroxides **C**. The obtained mercurylalkyl peroxides **C** can be demercurated using sodium borohydride or by bromonolysis. Both peroxymercuration and demercuration occur rapidly under mild conditions.

**Scheme 39:**
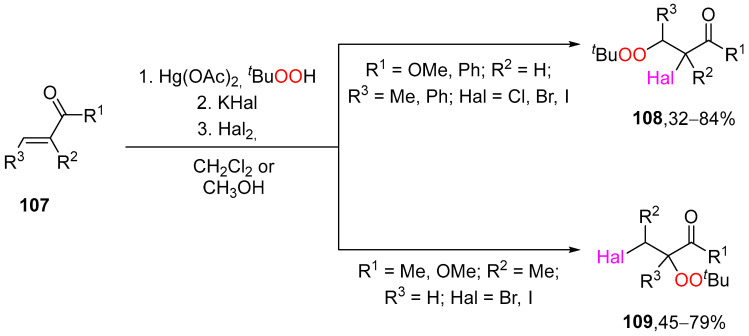
Synthesis of peroxides **108**, **109**.

Bloodworth A.J. demonstrated a two-stage approach to halogeno-alkyl peroxides **108**, **109** (Scheme 39) [131]. At the first stage, peroxymercuration of such unsaturated ketones **107** was carried out with the use of *^t^*BuOOH/Hg(OAc)_2_ system then demercuration of peroxymercurated product afforded with the formation of target peroxides **108**, **109** in 32–84% and 45–79% yields, respectively.

**Scheme 40:**
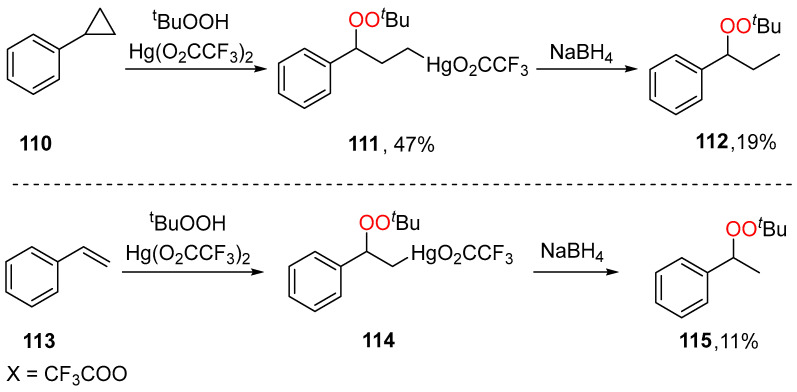
Synthesis of alkyl peroxides **112** and **115**.

Phenyl cyclopropane **110** undergoes ring opening under the action of the *^t^*BuOOH/Hg(CF_3_COO)_2_ system with the formation of mercurylalkyl peroxide **111** in a 47% yield. Further reduction **111** leads to alkyl peroxide **112** in a 19% yield [132]. The same system was applied for the synthesis of peroxide **115** from styrene **113** (Scheme 40).

**Scheme 41:**
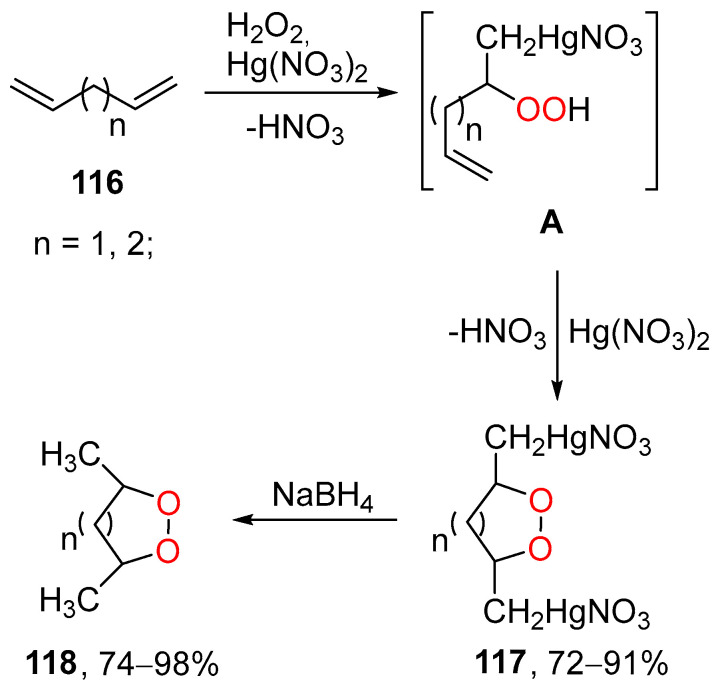
Synthesis of cyclic peroxides **117** and **118**.

In 1976, Bloodworth A. J. and colleagues described a method for the synthesis of cyclic peroxides **117** by the peroxymercuration of non-conjugated acyclic dienes **116**. The demercuration of **117** under the action of NaBH_4_ led to peroxides **118** (Scheme 41) [133].

**Scheme 42:**
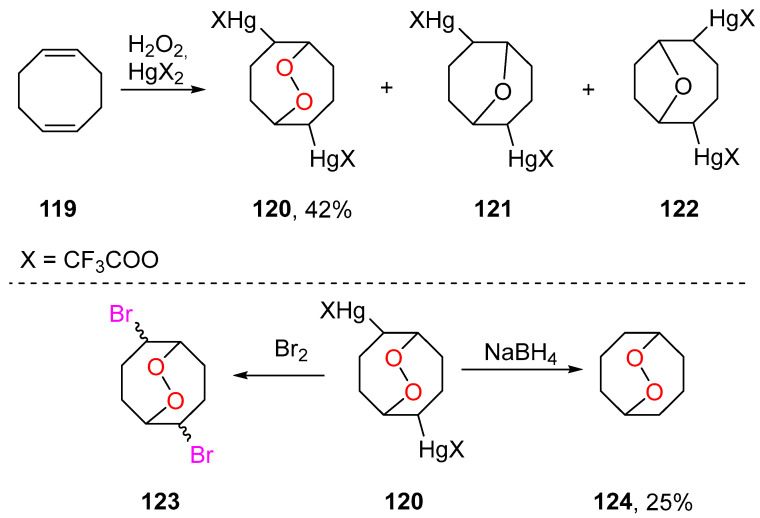
Synthesis of bicyclic peroxides **120**, **123** and **124**.

Adam W. et al. presented a method for the regioselective synthesis of bicyclic peroxide **120** by the peroxymercuration of non-conjugated cyclic dienes **119** (Scheme 42) [134]. Organo-mercury trifluoroacetates were separated by dissolving their mixture in benzene. The peroxide **120** did not dissolve in benzene and precipitated as white crystals. Reductive demercuration of **120** proceeded under mild conditions with the formation of bridged 1,2-dioxepane **124**. Bromination of peroxide **120** followed by demercuration led to dibromocycloperoxide **123**.

**Scheme 43:**
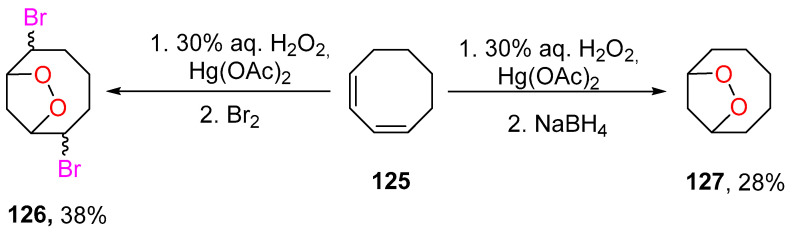
Synthesis of bicyclic peroxides **126** and **127**.

The peroxymercuration and demercuration of 1,4-cyclooctadiene **125** proceeded in a similar way with the formation of peroxides **126** and **127** (Scheme 43). Peroxides **126** and **127** were obtained in 38% and 28% yield respectively [135].

**Scheme 44:**
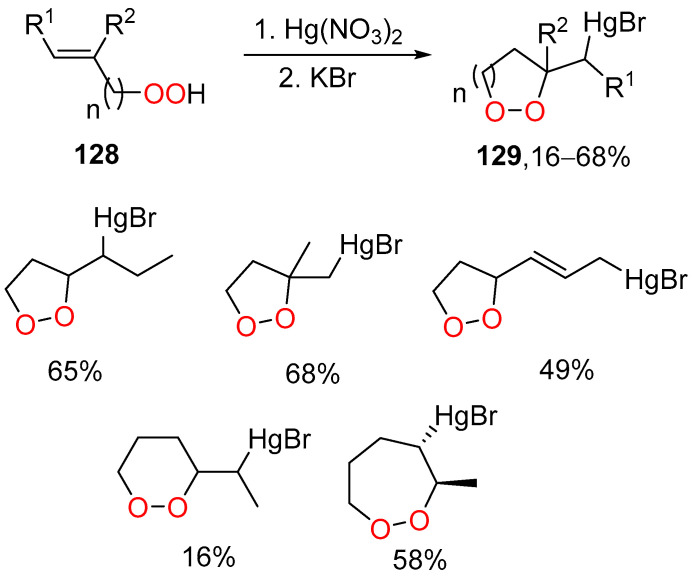
Synthesis of cyclic peroxides **129**.

Hydroperoxides **128** in the presence of mercury (II) nitrate undergo intramolecular cyclization with the formation of cyclic peroxides **129** in a yield of 16 to 68%. (Scheme 44) [136].

**Scheme 45:**
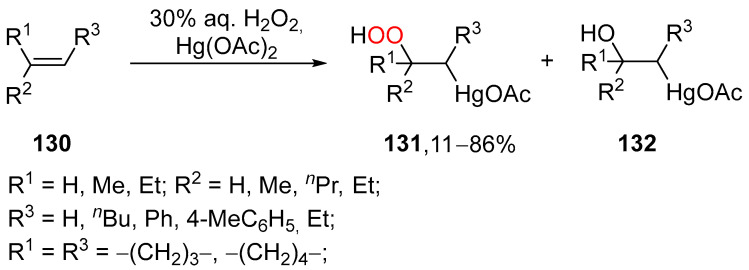
Synthesis of hydroperoxide **131**.

Hydroperoxymercuration of alkenes **130** with the use of aq. H_2_O_2_ proceeds with the formation of hydroperoxide **131** and alcohol **132**. The resulting peroxides **131** were obtained in yield up to 86% (Scheme 45) [137,138].

**Scheme 46:**
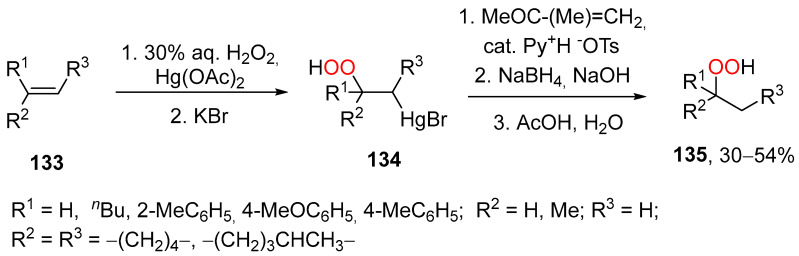
Synthesis of hydroperoxides **134** and **135**.

Direct demercuration of peroxides **134** is not possible because the hydroperoxide group is reduced under the action of sodium borohydride. However, the subsequent protection of hydroperoxy group by 2-methoxypropene, borohydride reduction, and deprotection of peroxy group led to peroxides **135** in 30–54% yield (Scheme 46) [138].

**Scheme 47:**
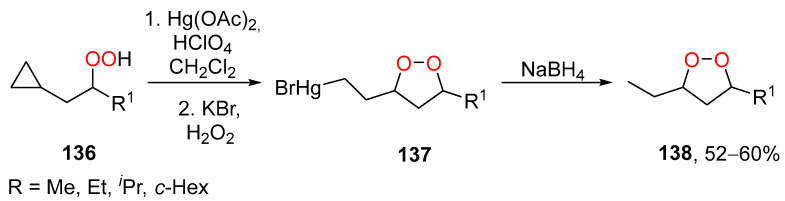
Synthesis of 1,2-dioxolane **138**.

Hydroperoxycyclopropanes **136** under the action of Hg(OAc)_2_ in the presence of perchloric acid were transformed into 1,2-dioxolanes **137**, the bromodemercuration of which led to 1,2-dioxolanes **138** (Scheme 47) [139]. Cyclic peroxides were isolated by column chromatography on SiO_2_ at 0 °C. The target peroxides **138** were obtained in 52–60% yield.

**Scheme 48:**
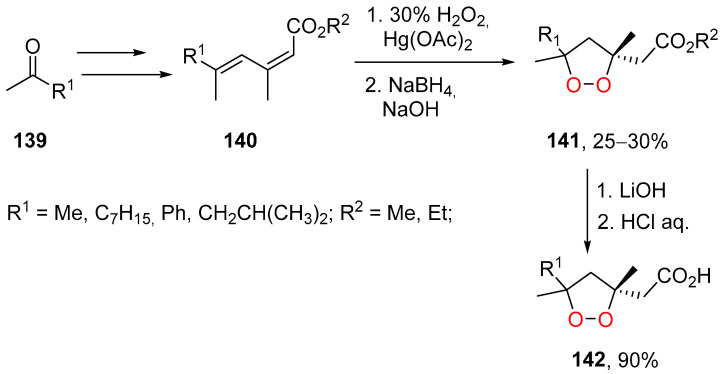
Synthesis of plakinic acids **142**.

The first example of the synthesis of diastereomeric saturated analogs of plakinic acids A, C and D **142** was described in 1996 by Bloodworth A. J. and colleagues [140]. Peroxides **142** were obtained in four stages from ketones **139**. At one stage of this synthetic route, the peroxymercuration of esters **140** was used with the formation of 1,2-dioxolanes **141**. Saponification of which led to 1,2-dioxolanes **142** with a free carboxyl group (Scheme 48).

### 2.5. Other Metal-Based Lewis Acids

**Scheme 49:**
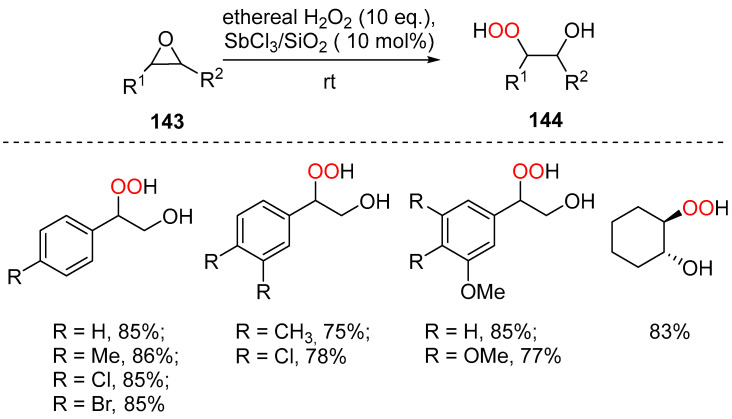
Synthesis of β-hydroperoxy alcohols **144**.

Zhang and Li reported the synthesis of β-hydroperoxy alcohols **144** by the reaction of epoxides **143** with H_2_O_2_, catalyzed by silica-supported antimony trichloride (SbCl_3_/SiO_2_) (Scheme 49) [141]. Interestingly, the authors demonstrated that SbCl_3_/SiO_2_ is more active than unsupported-SbCl_3_. Under the best conditions, a range of β-hydroxy hydroperoxides **144** was obtained in 72–86% isolated yields.

**Scheme 50:**
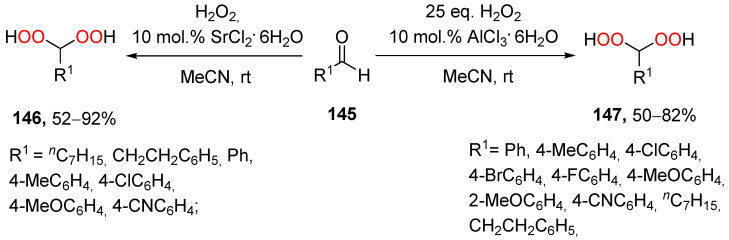
Synthesis of geminal bishydroperoxides **146** and **147** from aldehydes **145**.

Azarifar D. et.al. developed a method for the synthesis of geminal bishydroperoxides **146** from aldehydes **145** and hydrogen peroxide under the action of AlCl_3_·6H_2_O (Scheme 50) [114]. SrCl_2_**·**6H_2_O can also be used as a catalyst for the transformation of aldehydes **145** to the corresponding geminal bishydroperoxides **147**. Both catalysts allow the synthesis of target peroxides **146** and **147** under mild conditions at room temperature in good yields (Scheme 50) [142].

**Scheme 51:**
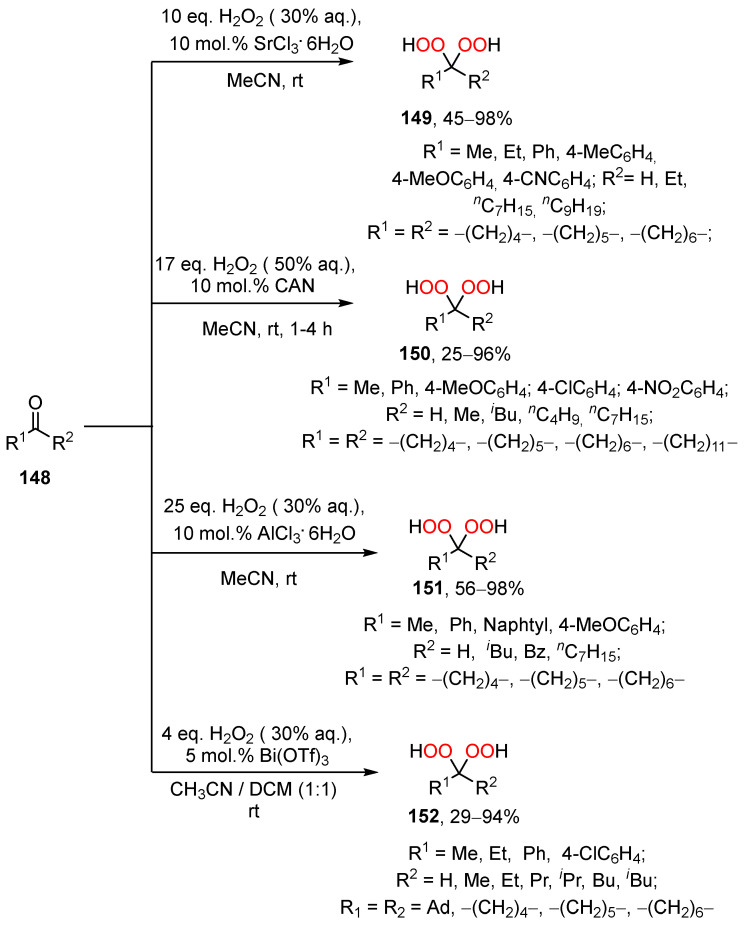
Synthesis of geminal bishydroperoxides **149**–**152**.

Lewis acids such as SrCl_3_**·**6H_2_O [142], cerium ammonium nitrate (CAN) [143], Bi(OTf)_3_ [144], and AlCl_3_·6H_2_O [114] are effective catalysts for the synthesis of bishydroperoxides **149**–**152** from cyclic and acyclic ketones and aldehydes **148**. Peroxidation proceeds under mild conditions at room temperature with the formation of target peroxides in a good yield. All Lewis acids demonstrated approximately equal efficiency in the peroxidation reaction. The main advantage of these methods is the use of Lewis acids in catalytic amounts and an inexpensive 30% aqueous H_2_O_2_ (Scheme 51).

**Scheme 52:**
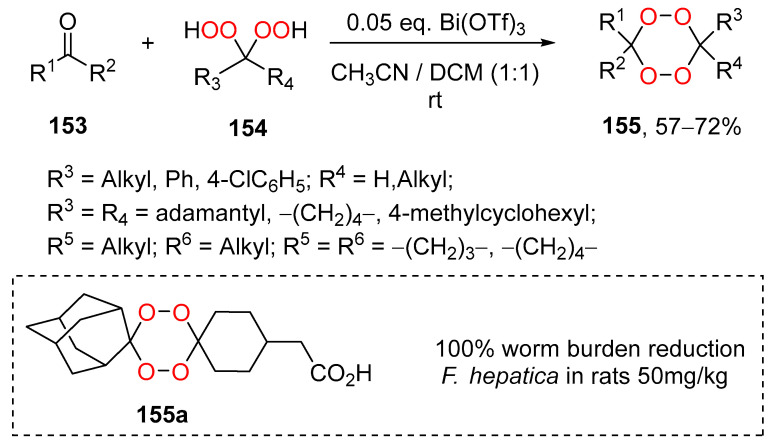
Synthesis of 1,2,4,5-tetraoxanes **155**.

Also, bismuth (III) triflate is a good catalyst for the synthesis of 1,2,4,5-tetraoxanes **155**. In this case, the target peroxides **152** were obtained in a yield up to 94%. Synthetic 1,2,4,5-tetraoxane **155a** exhibits high activity against helminths *Fasciola hepatica* and in rats in vivo (Scheme 52) [144,145].

**Scheme 53:**
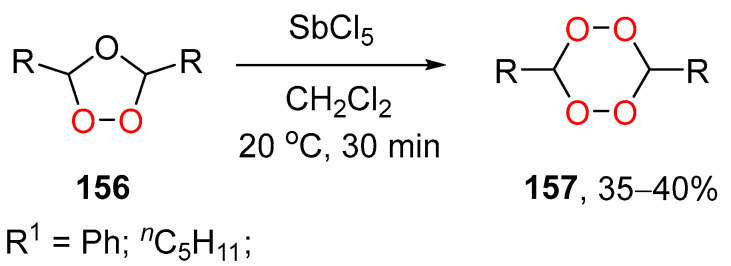
Synthesis of 1,2,4,5-tetraoxanes **157** from 1,2,4-trioxolanes **156**.

The interaction of 1,2,4-trioxolanes (ozonides) **156** with Lewis acid SbCl_5_ in methylene chloride led to 1,2,4,5-tetraoxanes **157** (Scheme 53) [146].

**Scheme 54:**
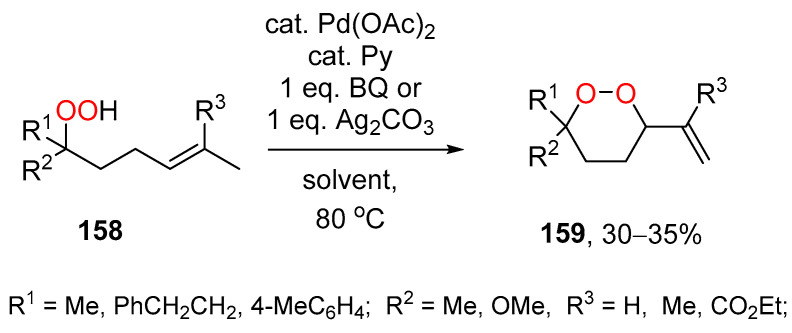
Synthesis of 1,2-dioxanes **159**.

The palladium-catalyzed cyclization of unsaturated hydroperoxides **158** afforded with the formation of 1,2-dioxanes **159** (Scheme 54) [147]. The reaction was carried out in toluene, 1,4-dioxane, or 1,2-dichloroethane at 80 °C for 3h. To oxidize Pd(0), which is formed in the catalytic cycle, *p-*benzoquinone (BQ) or silver carbonate were used.

**Scheme 55:**
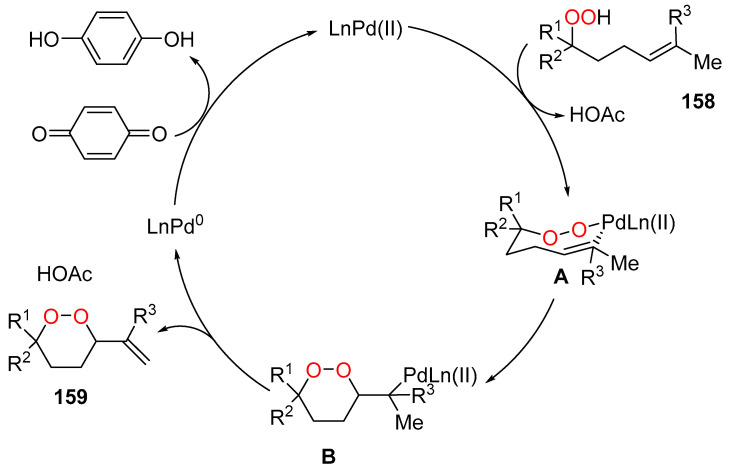
Synthesis of 1,2-Dioxanes **159** from hydroperoxides **158**.

Presumably, the reaction proceeds according to the characteristic Pd-catalyzed cycle, which is demonstrated in Scheme 55. Pd (II) is coordinated both with the double bond and the peroxide group to form cyclic intermediate **A**, which is further rearranged into endoperoxide **B**. Then endoperoxide **B** is converted to the target product **159**.

**Scheme 56:**
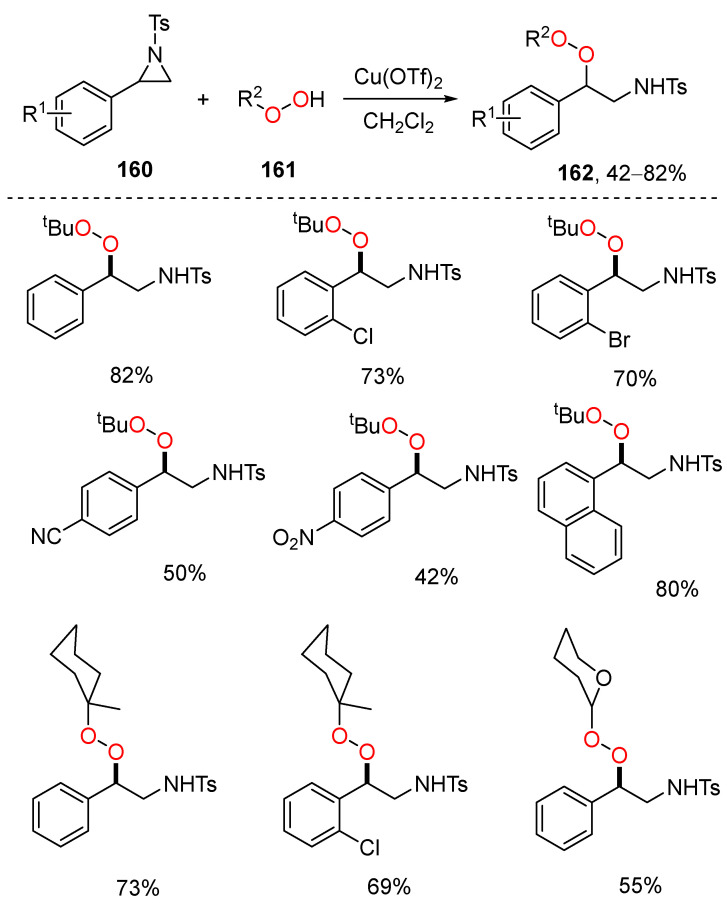
Synthesis of peroxides **162** from substituted aziridines **160**.

Such a Lewis acid as Cu(OTf)_2_ turned out to be the most effective catalyst for the synthesis of peroxides **162** by the ring opening reaction of activated aziridines **160** under the action of various hydroperoxides **161**. It was found that electron-neutral or halogenated substrates **160** provide better results in comparison with substrates containing electron-withdrawing substituents in an aromatic ring (Scheme 56) [126].

## 3. Non-Metal-Based Lewis Acids in the Synthesis of Organic Peroxides

There is great interest in Lewis acids based on non-metals. Their use as a catalyst or reagent made it possible to discover new classes of peroxides of various structures. This section contains data on the synthesis of 1-hydroperoxy-1′-alkoxyperoxides, β-hydroperoxy-β-peroxylactones, 1,2-dioxanes, 1,2,4-trioxepanes, 1,2,4-trioxocanes, 1,2,4-trioxonanes and 1,2,4,5,7,8-hexaoxananes.

### 3.1. Application of BF_3_·Et_2_O in the Synthesis of Organic Peroxides

**Scheme 57:**
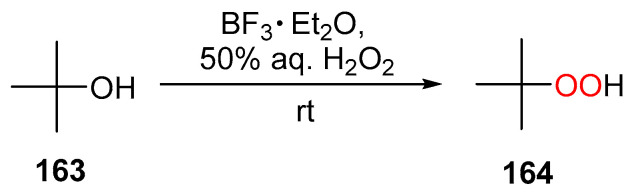
Synthesis of *tert*-butyl hydroperoxide **164**.

The first mentions of the formation of peroxides under the action of boron trifluoride goes back to the 1950s. A US patent 2,630,456 [148] from 1953 describes a selective method for producing *tert*-butyl hydroperoxide **164** from the corresponding alcohol **163** [149]. The reaction was carried out at room temperature using an equimolar amount of a 50% aqueous solution of hydrogen peroxide with 0.3 eq. of boron trifluoride etherate (Scheme 57). Since BF_3_ can form BF_3_·H_2_O complex [150,151,152,153,154,155], this makes it possible to use BF_3_·Et_2_O in the presence of water.

**Scheme 58:**
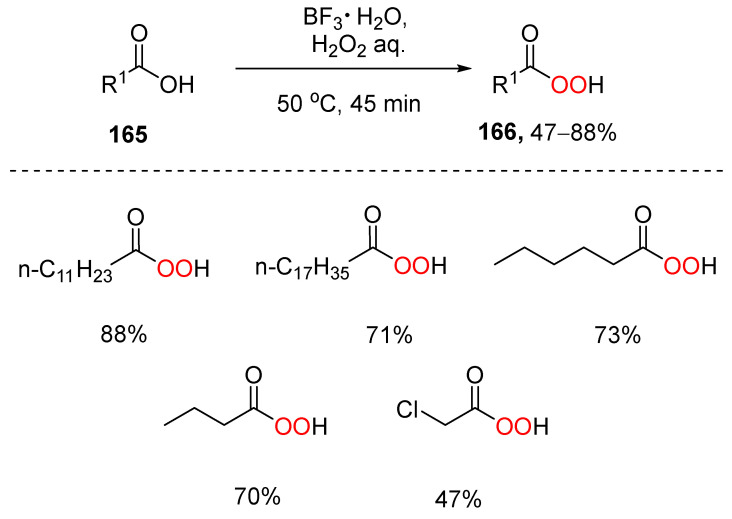
Synthesis of peroxy acids **166**.

In 1956, a method was developed for the synthesis of peroxy acids **166** with the use of boron trifluoride [156]. The synthesis was based on the interaction of a 90% aq. solution of hydrogen peroxide with carboxylic acids **166** in the presence of boron trifluoride monohydrate. The reaction was carried out for 45 min at 50 °C (Scheme 58). This approach was used for the synthesis of butyric, nylon and α-chloroacetic peroxy acids.

**Scheme 59:**
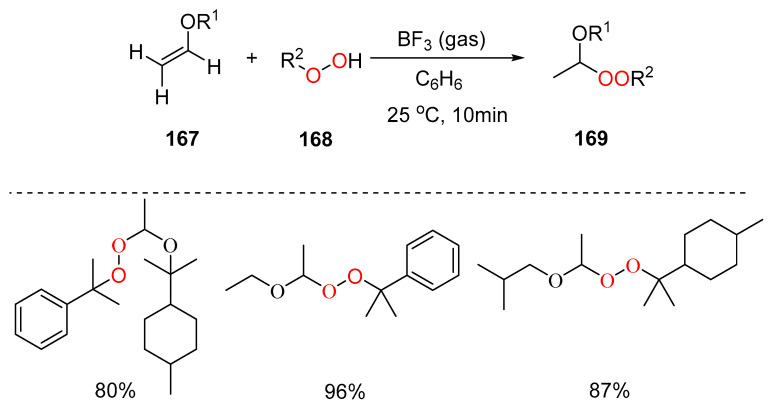
Synthesis of peroxyacetals **169** from vinyl esters **167**.

The reaction of vinyl esters **167** and hydroperoxides **168** in the presence of gaseous boron trifluoride leads to the formation of monoperoxyketals **169**. The reaction was carried out in benzene or hexane at temperatures from 0 to 30 °C (Scheme 59) [157]. The reaction proceeds within 5–10 min with a yield of 80–96%. This method is the first way to obtain monoperoxyacetals in high yields.

**Scheme 60:**
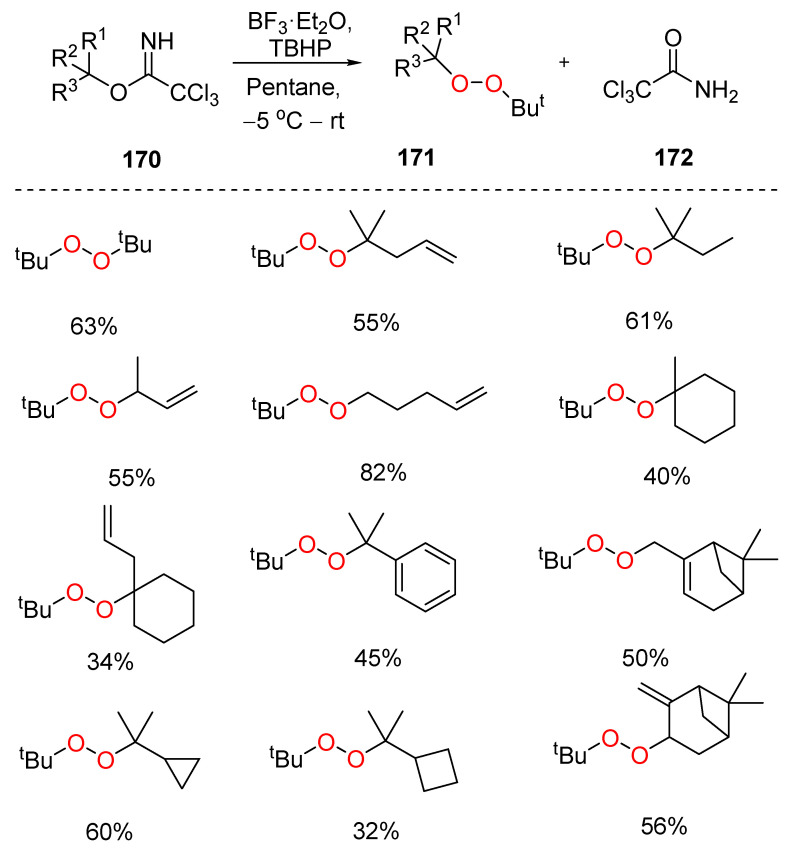
Synthesis of alkyl peroxides **171**.

The synthesis of alkyl peroxides **171** was carried out by the reaction of tertiary alkyl-trichloroacetimides **170** with *tert*-butyl hydroperoxide in the presence of boron trifluoride etherate (Scheme 60) [158].

**Scheme 61:**
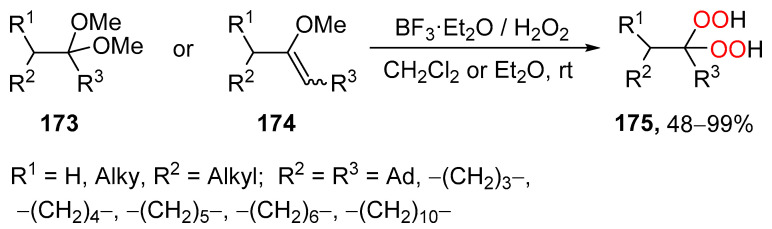
Synthesis of bishydroperoxides **175**.

A wide range of bishydroperoxides **175** was obtained from acetals **173**, enol ethers **174** and hydrogen peroxide in the presence of boron trifluoride etherate (Scheme 61) [159,160]. The developed method allows one to obtain peroxides of various structures. The advantages of these reactions are the rapidity and ease of its implementation, and among the disadvantages can be noted the formation of by-products, as well as the impossibility of synthesizing bishydroperoxides from acetals or enol ethers obtained from aryl-substituted ketones.

**Scheme 62:**
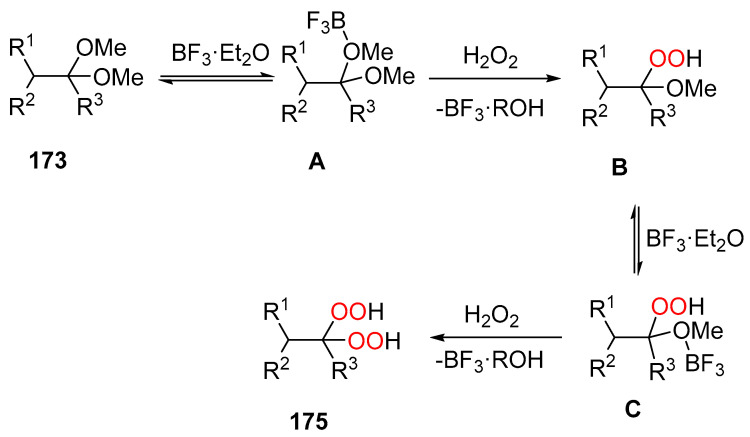
Mechanism of acetal peroxidation **173**.

Presumably, the reaction proceeds according to the following mechanism: BF_3_·Et_2_O and BF_3_·MeOH equally catalyze the reaction, forming intermediate complexes **A** and **B**, which then interact with hydrogen peroxide with the formation of bishydroperoxide **175** (Scheme 62).

**Scheme 63:**
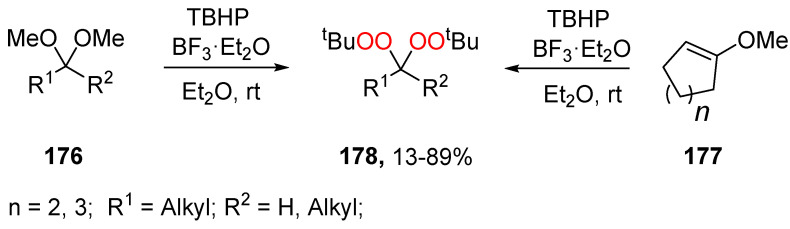
Synthesis of germinal bis(*tert*-butyl) peroxides **178**.

The possibility to obtain geminal bis(*tert*-butyl)peroxides **178** of both cyclic and acyclic structures with a yield of 13% to 89%, respectively, was described from acetals **176** and enol ethers **177** (Scheme 63) [161]. The reaction of the enol esters **177** with *tert*-butyl hydroperoxide, catalyzed by boron trifluoride etherate, is a general approach for the preparation of geminal bishydroperoxides.

**Scheme 64:**
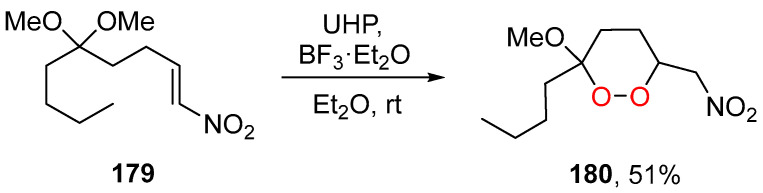
Synthesis of 1,2-dioxane **180**.

1,2-Dioxane **180** was obtained by the reaction of the corresponding acetal **179** with urea hydrogen peroxide, catalyzed by boron trifluoride etherate (Scheme 64) [162]. Under these conditions, only one of the two methoxyl groups is exchanged for the hydroperoxide one, and the intermediate hydroperoxyketal undergoes intramolecular cyclization (according to Michael) due to the attack of the hydroperoxide group on the double bond activated by the nitro group with the formation of 1,2-dioxane in 51% yield.

**Scheme 65:**
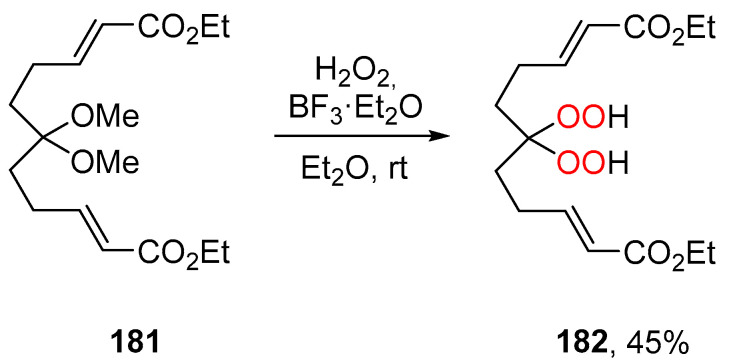
Synthesis of bishydroperoxide **182**.

However, when NO_2_ was replaced by C(O)OEt, the reaction proceeded with the formation of bisperoxide **182** (Scheme 65) [163]. This is probably due to the fact that the ester group has lower electron-withdrawing properties.

**Scheme 66:**
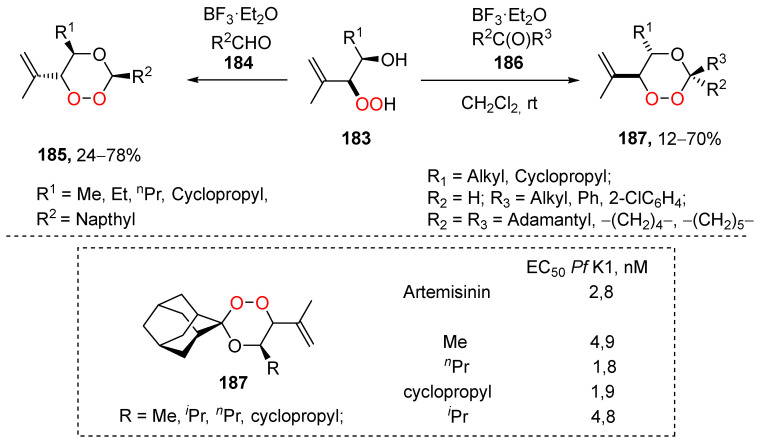
Synthesis of 1,2,4-trioxanes **185** and **187**.

An efficient and stereoselective method for the synthesis of 1,2,4-trioxanes **185** and **187** has been reported by J.L. Vennerstrom (Scheme 66). Such peroxides were obtained by the interaction of α-hydroxyperoxides **183** with aldehydes **184** and ketones **186** [52,164,165,166,167]. The resulting cyclic peroxides were tested in vitro for antimalarial activity against *P. falciparum*. 1,2,4-Trioxanes **187** containing an adamantane substituent in their composition exhibit high antimalarial activity.

**Scheme 67:**
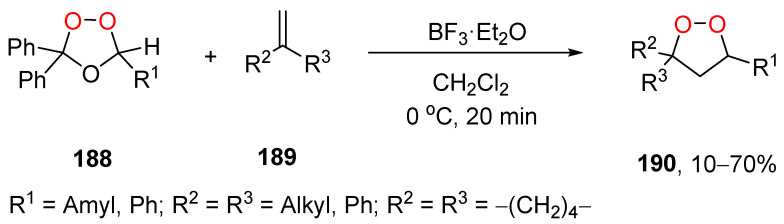
Synthesis of 1,2-dioxolanes **190**.

An unusual method for the synthesis of 1,2-dioxolanes **190** was developed, which is based on the reaction of ozonides **188** with olefins **189** in the presence of boron trifluoride etherate with a yield of 10% to 70% (Scheme 67) [168].

**Scheme 68:**
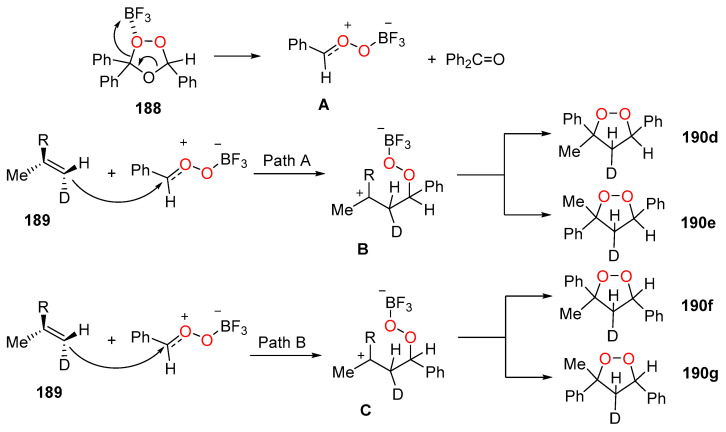
The proposed mechanism of 1,2-dioxolanes **190** formation.

Presumably, the reaction proceeds along the following route: the first stage of the reaction involves the opening of the ozonide cycle in **188** under the action of BF_3_·Et_2_O with the formation of a BF_3_-coordinated intermediate **A**, containing a peroxide fragment. The attack of intermediate **A** at the alkene **189** is accompanied by the formation of two intermediates, **B** and **C**, which, in turn, leads to ring closure and gives 1,2-dioxolane. However, the rate of ring closure is much slower than the rotation of the C-C bond, so the formation of four isomeric products occurs. The mechanism in Scheme 68 illustrates that the ratio of (**190d** + **190e**) to (**190f** + **190g**) corresponds to the ratio of the two approaches of BF_3_-coordinated intermediate **A** to alkene.

**Scheme 69:**
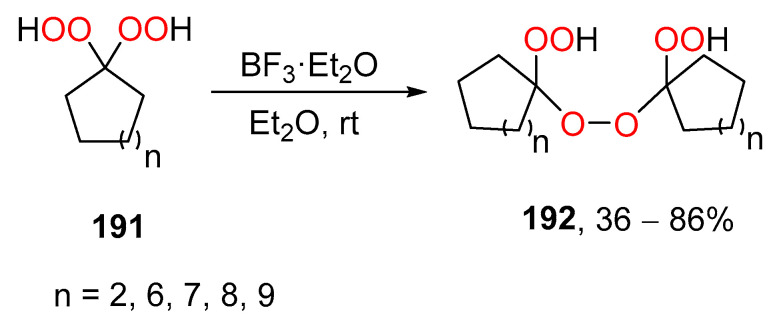
Synthesis of 1,1′-bishydroperoxy(cycloalkyl)peroxide **192**.

BF_3_·Et_2_O is an efficient catalyst for the synthesis of 1,1′-bishydroperoxy-(cycloalkyl) peroxides **192** from geminal bisperoxides **191** in yields of up to 86% (Scheme 69) [89].

**Scheme 70:**
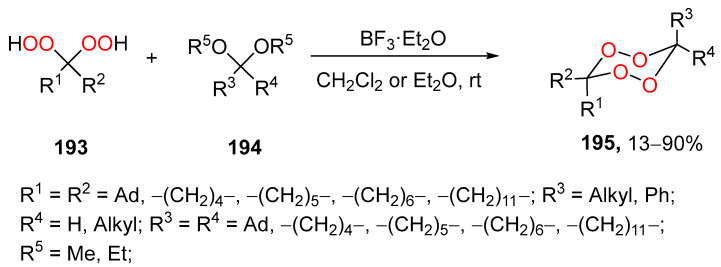
Synthesis of 1,2,4,5-tetraoxanes **195**.

Terent’ev et al. developed a method for the synthesis of 1,2,4,5-tetraoxanes **195** from bishydroperoxides **193** and acetals **194** (Scheme 70) [169]. The reaction was carried out under mild conditions at room temperature using 0.3–0.4 eq. BF_3_·Et_2_O. This method is general to the synthesis of unsymmetrical tetraoxanes from readily available carbonyl compounds.

**Scheme 71:**
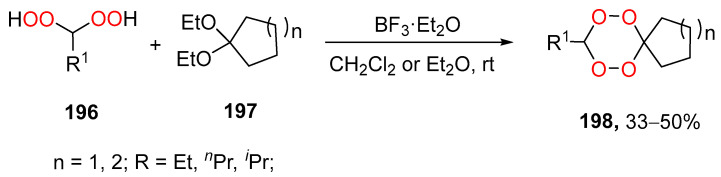
Synthesis of asymmetrical 1,2,4,5-tetraoxanes **198**.

Unsymmetrical tetaraoxanes **198** can be obtained from geminal bisperoxides **196** and cyclic acetals **197** in the presence of boron trifluoride etherate (Scheme 71) [170]. This method for the synthesis of 1,2,4,5-tetraoxanes is a convenient and simple approach to the synthesis of both symmetric and asymmetrical 1,2,4,5-tetraoxanes.

**Scheme 72:**
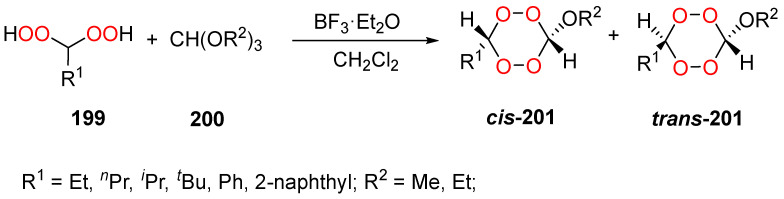
Synthesis of 1,2,4,5-tetraoxanes ***cis*-201** and ***trans*-201**.

Also, boron trifluoride etherate is efficient for the synthesis of 1,2,4,5-tetraoxanes **201** from gem-bisperoxides **199** and orthoformates **200** (Scheme 72) [170]. The *trans*-isomer **201** was the major product in all cases as determined by NMR, while the *cis*-isomer was found only in trace amounts. The reaction was carried out in dichloromethane at room temperature. This approach was the first method for the preparation of tetraoxanes ***cis-*201** and ***trans-*201** with an alkoxy substituent.

**Scheme 73:**
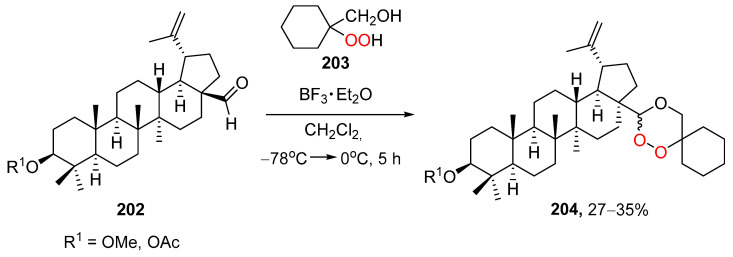
Synthesis of 1,2,4-trioxanes **204** from aldehydes **202**.

In the study on the synthesis of pharmacologically important endoperoxides, peroxide **204** was synthesized from substituted aldehydes **202**; boron trifluoride etherate was used as a catalyst in this reaction. Condensation of peroxide **203** with betulin aldehydes **202** in the presence of BF_3_∙Et_2_O led to the assembly of peroxides **204**. The yield of the target peroxide was low, and the resulting diastereoisomers could not be separated. Unfortunately, mixtures of isomers did not show significant anticancer activity (Scheme 73) [171].

**Scheme 74:**
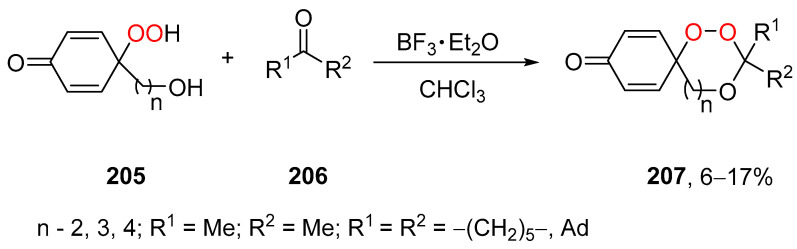
Synthesis of macrocyclic peroxides **207**.

Cyclic peroxides **207** can be obtained from hydroperoxides **205** and ketones **206** in the presence of boron trifluoride etherate in up to 17% yield (Scheme 74) [130]. The yield of the target peroxides **207** was in the same range as when using In(OTf)_3_ as a catalyst (see Scheme 33). However, BF_3_·Et_2_O is less expensive than In(OTf)_3_.

**Scheme 75:**
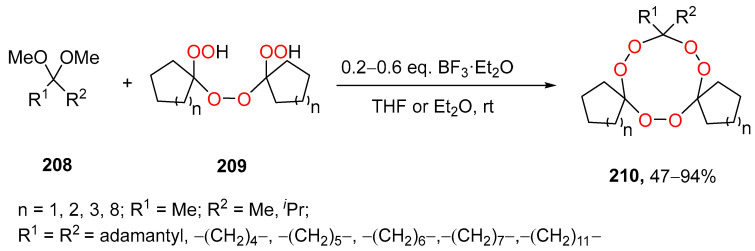
Synthesis of 1,2,4,5,7,8-hexaoxonanes **210**.

The reaction of 1,1′-bishydroperoxy(cycloalkyl)peroxides **209** with ketals **208** in the presence of BF_3_·Et_2_O afforded 1,2,4,5,7,8-hexaoxonanes **210** in up to 94% yields (Scheme 75) [172]. This approach is convenient and simple for the synthesis of 1,2,4,5,7,8-hexaoxonanes, which significantly expands the structural diversity of these compounds and, in most cases, allows them to be synthesized in high yield.

**Scheme 76:**
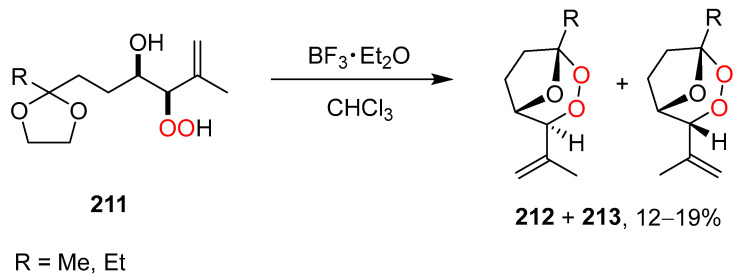
Synthesis of bridged 1,2,4-trioxanes **212** and **213**.

The assembly of bridged 1,2,4-trioxanes **212** and **213** can be accomplished by intramolecular cyclization of peroxyacetals **211** with simultaneous removal of the acetal protecting group. Cyclic peroxides were obtained in a yield of 12% to 19% (Scheme 76) [173].

**Scheme 77:**
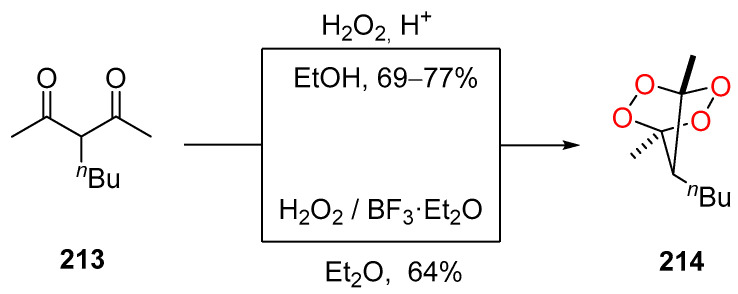
Synthesis of bridged 1,2,4,5-tetraoxane **214**.

A convenient, experimentally simple and selective method was developed for the synthesis of bridged 1,2,4,5-tetraoxanes based on the reaction of hydrogen peroxide with β-diketone **213** catalyzed by strong acids (H_2_SO_4_, HClO_4_, HBF_4_) with a yield of 49–77% (Scheme 77) [37,76]. This process can also proceed with the use of Lewis acid (BF_3_·Et_2_O). For example, tetraoxane **214** was obtained in 64% yield [76].

**Scheme 78:**
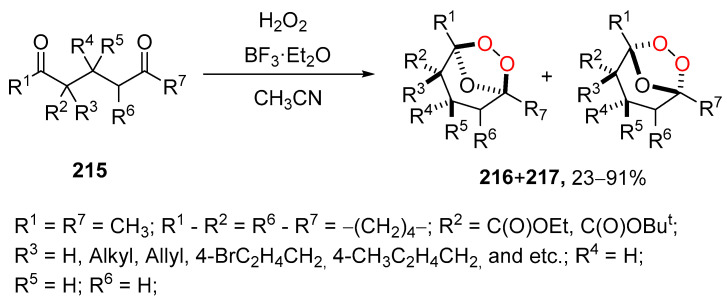
Synthesis of diastereomeric ozonides **216** and **217** from 1,5-diketones **215**.

The method for the synthesis of ozonides **216** and **217** from 1,5-diketones **215** and hydrogen peroxide, which does not require the use of toxic ozone, was reported (Scheme 78) [84,174]. It was found that the interaction of 1,5-diketones **215** with H_2_O_2_, in the presence of BF_3_·Et_2_O leads to the selective assembly of stereoisomeric ozonides **216** and **217**. Peroxides **216** and **217** exhibit antimalarial [175] and anticancer [175,176] activity.

**Scheme 79:**
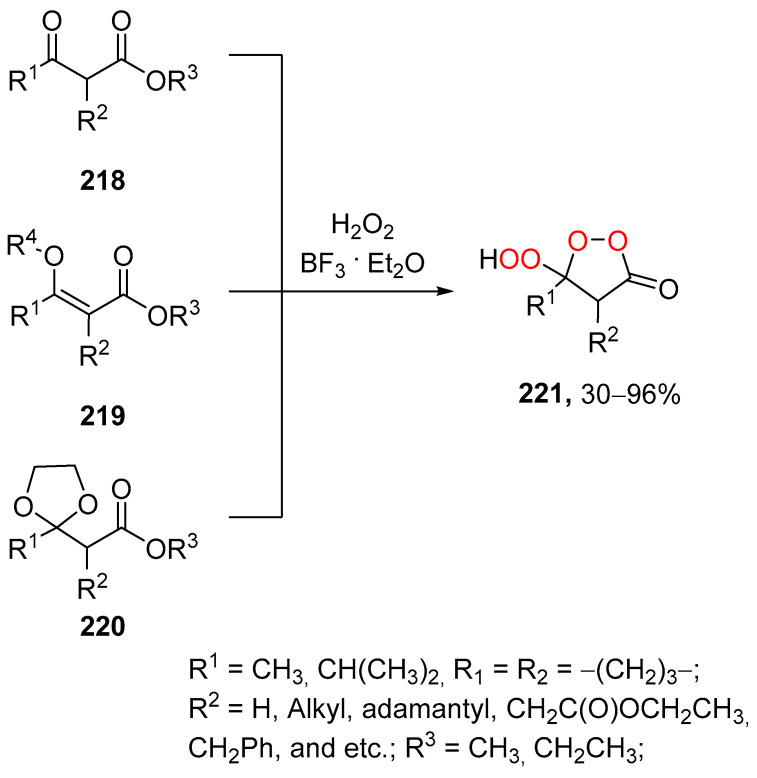
Synthesis of β-hydroperoxy-β-peroxylactones **221**.

Recently a new class of peroxides, namely β-hydroperoxy-β-peroxylactones **221,** was discovered. They were obtained by the peroxidation of β-ketoesters **218** and their derivatives **219** and **220** (silylenol ethers, alkylene ethers, enol acetates, cyclic acetals) with the H_2_O_2_/BF_3_·Et_2_O system. The reaction proceeded with the formation of β-hydroperoxy-β-peroxylactones in a yield of 30–96% (Scheme 79) [85,177]. These β-peroxylactones are stable and can be useful for further synthetic transformations.

**Scheme 80:**
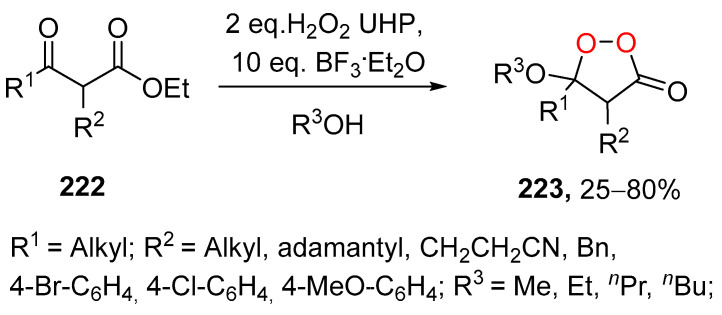
Synthesis of β-alkoxy-β-peroxylactones **223**.

The three-component cyclization/condensation of β-ketoesters **222**, H_2_O_2_ UHP, and alcohols proceeded with the formation of β-alkoxy-β-peroxylactoctones **223** in 25–80% yield (Scheme 80) [178].

**Scheme 81:**
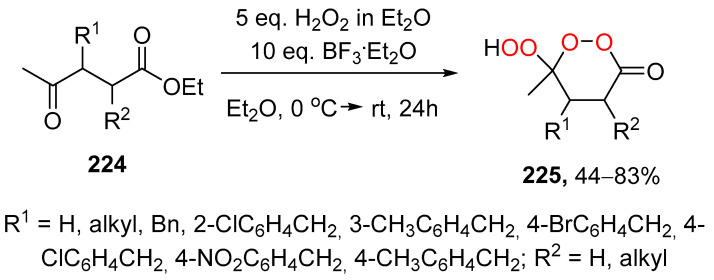
Synthesis of γ-alkoxy-γ-peroxylactones **225**.

In continuation of studies in this direction, the BF_3_∙Et_2_O/H_2_O_2_ system was applied to the γ-ketoesters **224**. Peroxidation proceeded with the formation of cyclic γ-hydroperoxy-γ-peroxylactones **225** in 44–83% yields (Scheme 81) [179].

**Scheme 82:**
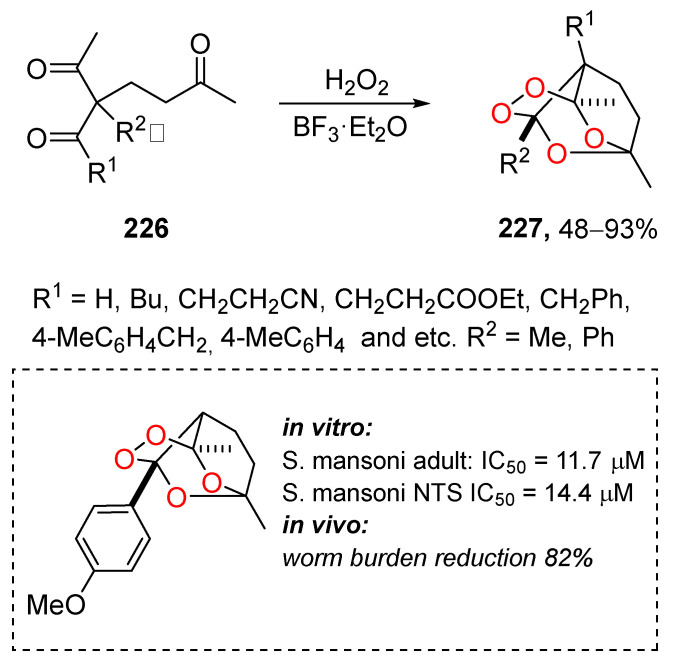
Synthesis and anthelmintic activity of tricyclic monoperoxides **227**.

Tricyclic monoperoxides **227** were obtained by the peroxidation of β,δ′-triketones **226** with the H_2_O_2_/BF_3_·Et_2_O system (Scheme 82) [81,86]. Peroxidation was carried out under mild conditions at room temperature for 1 h. Despite the presence of three carbonyl groups, peroxidation proceeded selectively with the formation of cyclic product **227**. The yield of target peroxides **227** was 48–93%. It was found that the tricyclic monoperoxide exhibits a high in vitro and in vivo anthelmintic activity against *S. mansoni*.

**Scheme 83:**
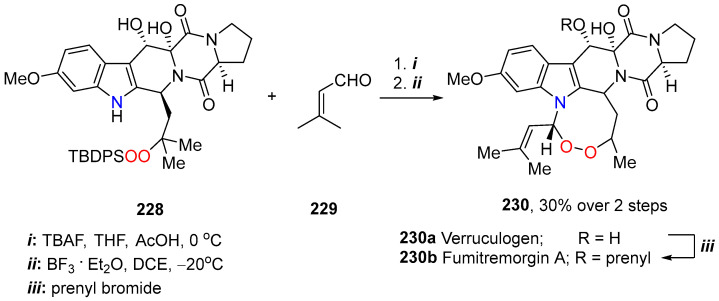
Synthesis of natural Verruculogen **230a** and Fumitremorgin A **230b**.

The first total synthesis of natural bioactive azaperoxides Verruculogen **230a** and Fumitremorgin A **230b** was developed in 2015 by the Baran group [180]. The final step included the catalyzed by BF_3_·Et_2_O condensation of aldehyde **229** with peroxide **228** (Scheme 83).

### 3.2. Iodine in the Synthesis of Organic Peroxides

**Scheme 84:**
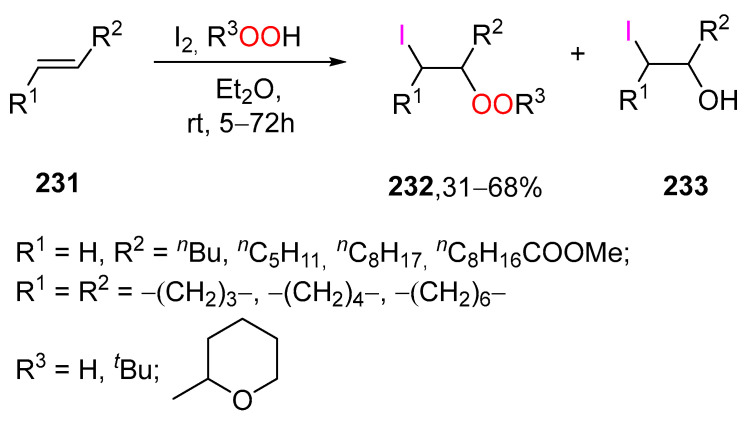
Synthesis of vicinal iodoperoxyalkanes **232**.

Iodine in the synthesis of organic peroxides can act as both a catalyst and a reagent. The presence of iodine can activate substrates via halogen bonding (acts as Lewis acid), iodonium(I) species or formation of “hidden” HI Broensted acid [181,182,183,184,185,186,187]. The interaction of alkenes **231** with hydroperoxide in the presence of molecular iodine makes it possible to obtain vicinal iodoperoxyalkanes **232** (Scheme 84) [188]. This reaction was carried out with 0.7 eq. iodine and 4 eq. hydroperoxide in diethyl ether or dichloromethane at room temperature. Depending on the reactivity of the hydroperoxide, the reaction time was from 5 to 72 h.

**Scheme 85:**
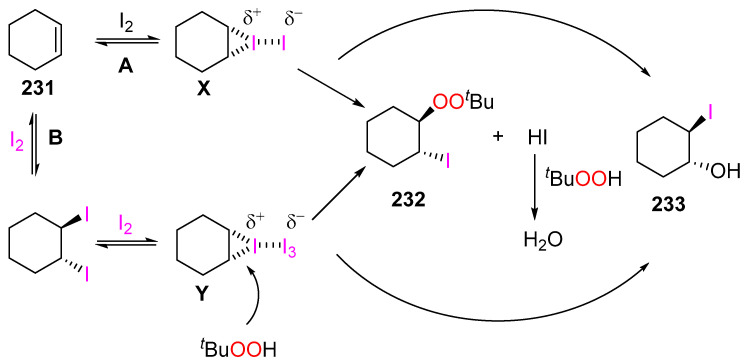
The mechanism of formation of iodoperoxyalkanes **232** and iodohydroxyalkanes **233**.

The mechanism of the formation of iodoperoxyalkanes and iodoalkanols is shown in Scheme 85. Presumably, the formation of iodoperoxyalkane can proceed along path **A** or **B**. Path **A** corresponds to the classical scheme of sequential addition of electrophilic iodine and nucleophilic hydroperoxide to the double bond. Path **B** is based on experimental data according to which an increase in the amount of iodine (a nucleophile competing with *tert*-butyl hydroperoxide) leads to an increase in the yield of 1-(*tert*-butylperoxy)-2-iodocyclohexane, while the expected 1,2-diiodocyclohexane is formed in trace amounts. Iodoperoxide appears to be formed by pathway **B** through a previously unknown process. Initially, the reaction forms 1,2-diiodocyclohexane, which is converted by iodine to intermediate **Y**, which contains a partially positive charge on the carbon atoms. The latter reacts with hydroperoxide.

**Scheme 86:**
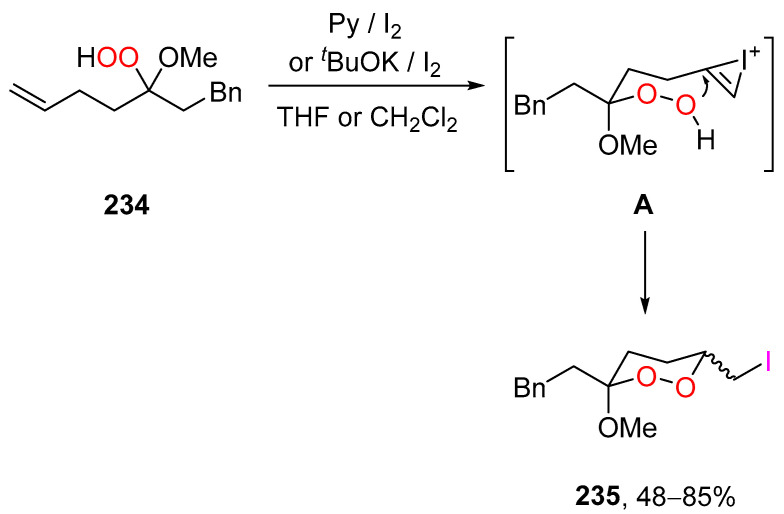
Cyclization of unsaturated alkoxy hydroperoxide **234**.

The cyclization of unsaturated hydroperoxyacetal **234** was performed using systems such as pyridine/I_2_ or *t*-BuOK/I_2_. The use of the latter made it possible to obtain 1,2-dioxanes **235** in a yield up to 85% (Scheme 86) [189].

**Scheme 87:**
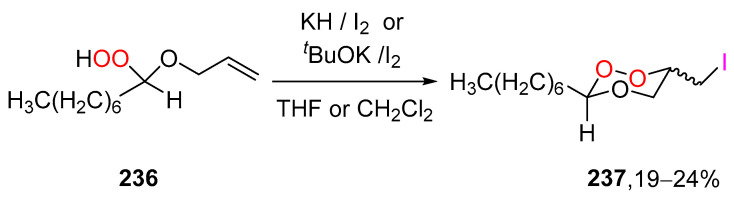
Synthesis of 1,2,4-trioxane **237**.

However, the use of the pyridine/I_2_ system for unsaturated hydroperoxyacetal **236** did not provide the assembly of 1,2,4-trioxane **237**. The *t*-BuOK/I_2_ system, which performed well in the assembly of 1,2-dioxalane **235** (Scheme 87), led to peroxide **237**, but in low yield. Cyclization **236** under the action of the KH/I_2_ system also proceeded in a low yield (Scheme 87) [189].

**Scheme 88:**
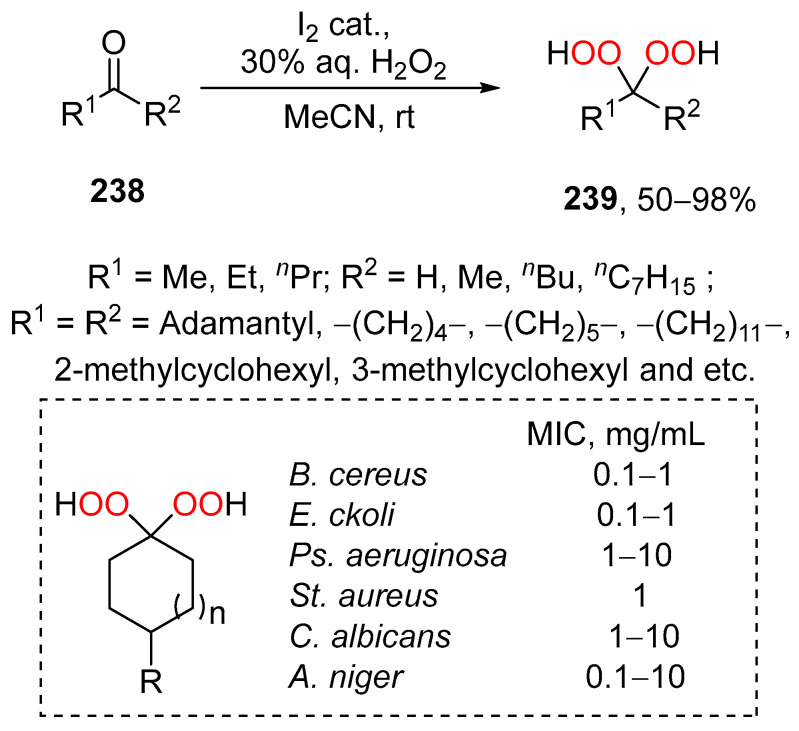
**S**ynthesis of geminal bishydroperoxides **239** and their activity.

Using 30% aq. H_2_O_2_ and iodine as a catalyst, geminal bishydroperoxides **239** were obtained from cyclic and acyclic ketones **238** in a yield of 50 to 98% (Scheme 88). All geminal bishydroperoxides **239** exhibit pronounced in vitro antimicrobial and antifungal activity against *B. cereus*, *E. coli*, *P. aeruginosa*, *S. aureus*, *C. albicans*, and *A. niger* [190].

**Scheme 89:**
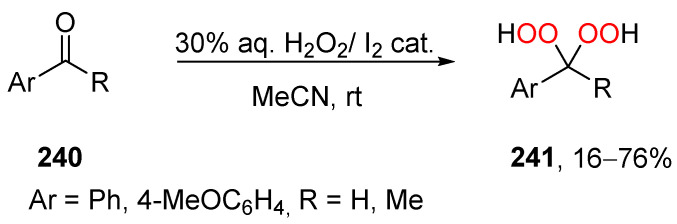
Synthesis of geminal dihydroperoxides **241**.

This approach was also used in the synthesis of bishydroxyperoxides **241** from acetophenone and benzaldehydes **240**. Unfortunately, peroxidation of compounds containing an electron-withdrawing substituent in the ring did not lead to the target geminal bishydroxyperoxides (Scheme 89) [190].

**Scheme 90:**
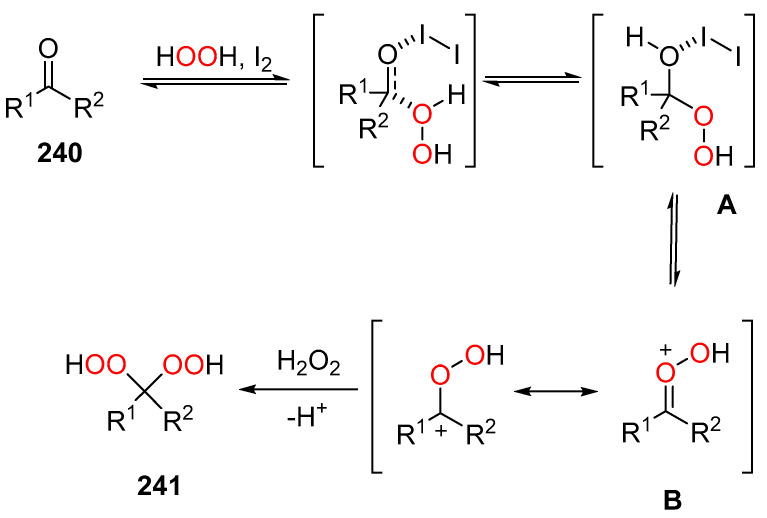
The proposed mechanism for the assembly of geminal bisperoxide **241**.

The action of iodine as a Lewis acid is based on its interaction with the oxygen atom of the carbonyl group of **240**, which facilitates the nucleophilic attack of hydrogen peroxide on the neighboring carbon atom. Iodine then eliminates the hydroxy group from the sp^3^-carbon atom of intermediate **A** and the peroxycarbenium ion **B** is formed, which is attacked by the second hydrogen peroxide molecule to form the final product **241**. The last stage of this mechanism is irreversible (Scheme 90).

**Scheme 91:**
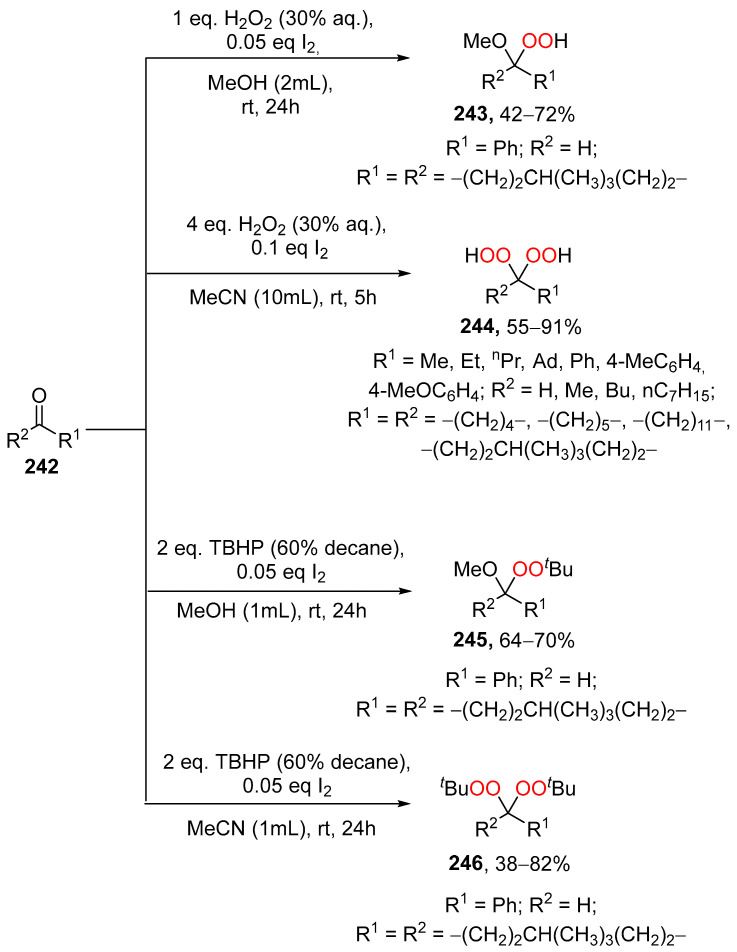
Iodine-catalyzed peroxidation of carbonyl compounds **242**.

The iodine-catalyzed peroxidation of carbonyl compounds **242** (acyclic and cyclic ketones and aromatic aldehydes), is a simple and effective approach to obtain geminal hydroperoxides **244** and geminal *tert*-butyl peroxides **246**. A similar reaction in methanol led to hydroperoxyacetals **243** and *tert*-butylperoxyacetals **245** (Scheme 91) [90].

**Scheme 92:**
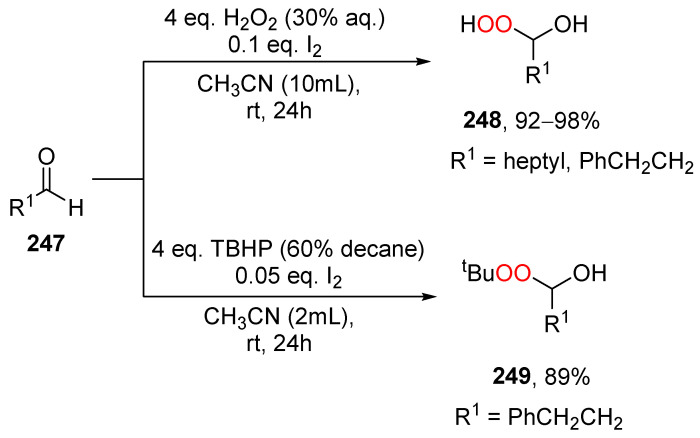
Iodine-catalyzed peroxidation of aldehydes **247**.

The application of I_2_/H_2_O_2_ and I_2_/TBHP systems to non-aromatic aldehydes allows one to obtain hydroxy-hydroperoxides **248** and *tert*-butylhydroxyperoxides **249** (Scheme 92) [190].

**Scheme 93:**
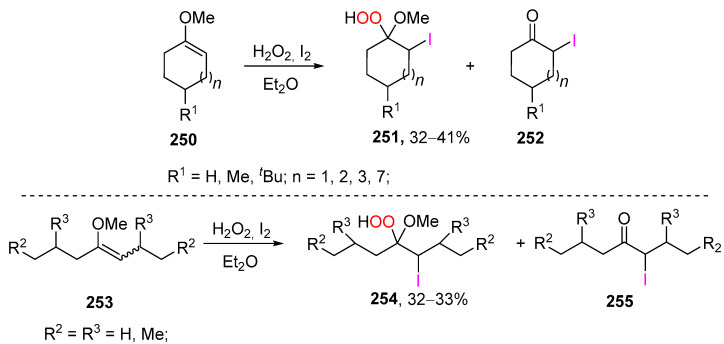
Synthesis of 2-iodo-1-methoxy hydroperoxides **251** and **254**.

Iodine-catalyzed peroxidation of enol ethers **250** and **253** in Et_2_O led to formation of 2-iodo-1-methoxy hydroperoxides **251** and **254**, respectively, with a yield of 32–41% (Scheme 93) [191]. α-Iodo ketones **252** and **255** were formed as byproducts.

**Scheme 94:**
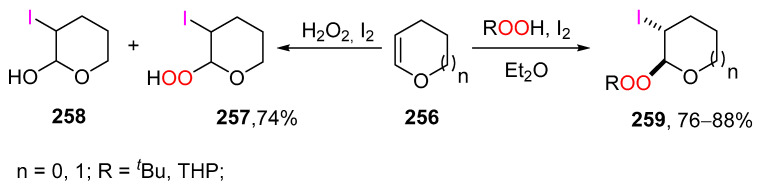
Synthesis of iodoperoxides **257** and **259**.

Peroxidation of monocyclic enol ethers **256** under the action of the I_2_/H_2_O_2_ system proceeded with the formation of iodo-hydroperoxides **257** and α-iodo hemiacetals **258**, while the reaction with I_2_/ROOH led only to iodoperoxides **259** (Scheme 94) [192].

**Scheme 95:**
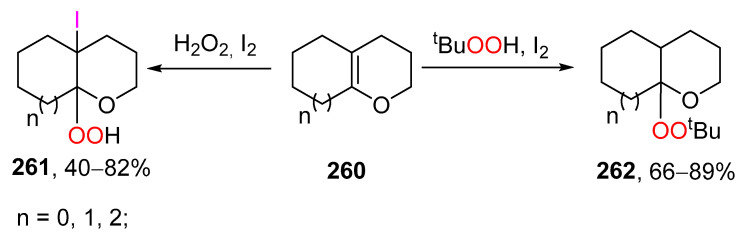
Synthesis of vicinal iodoperoxides **261** and peroxides **262**.

Bicyclic enol esters were converted to vicinal iodoperoxides **261** under the action of I_2_/H_2_O_2_ system in 40–82% yield. However, the use of *t*-BuOOH instead of H_2_O_2_ led to the formation of peroxides **262** without iodine in their composition with a yield of 66–89% yield (Scheme 95) [192].

**Scheme 96:**
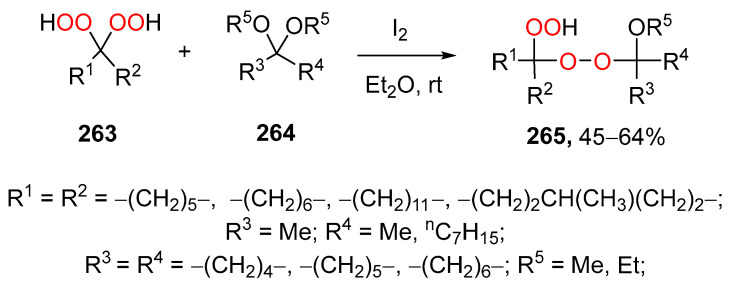
Synthesis of 1-hydroperoxy-1′-alkoxyperoxides **265**.

The previously unknown 1-hydroperoxy-1′-alkoxyperoxides **265** were synthesized in 45–64% yield by iodine-catalyzed reaction of geminal bishydroperoxides **263** with acetals **264** (Scheme 96) [193]. The nature of the solvent has a decisive influence on the yield of the target peroxides. Good results were obtained in such solvents as Et_2_O and THF. The formation of cyclic peroxides was not observed. 1-Hydroperoxy-1′-alkoxyperoxides **265** were readily isolated from the reaction mixture by column chromatography.

**Scheme 97:**
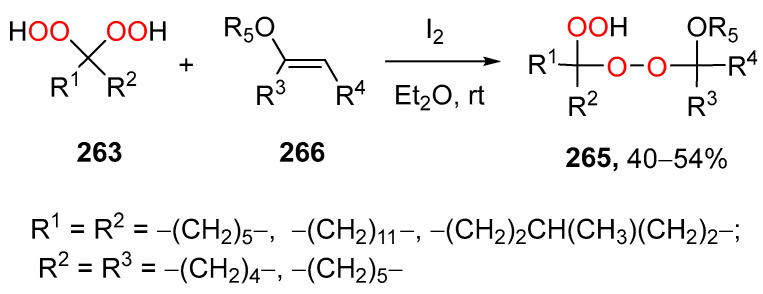
Synthesis of 1-hydroperoxy-1′-alkoxyperoxides **265**.

Also, 1-hydroperoxy-1′-alkoxyperoxides **265** are formed by the interaction of bishydroperoxides **263** with enol ethers **266** in the presence of molecular iodine (Scheme 97) [193].

**Scheme 98:**
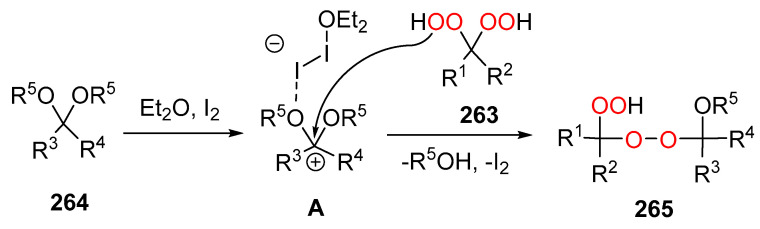
The proposed mechanism for the assembly of 1-hydroperoxy-1′-alkoxyperoxides **265**.

Initially, iodine, which is probably in the form of a complex with diethyl ether (or tetrahydrofuran), interacts with the oxygen atom of the methoxy group of acetal **264**. Then the geminal bishydroperoxide **263** attacks the electrophilic center that is formed at the quaternary carbon atom **A**. Finally, the elimination of methanol proceeds with the formation of target peroxide **265** (Scheme 98).

**Scheme 99:**
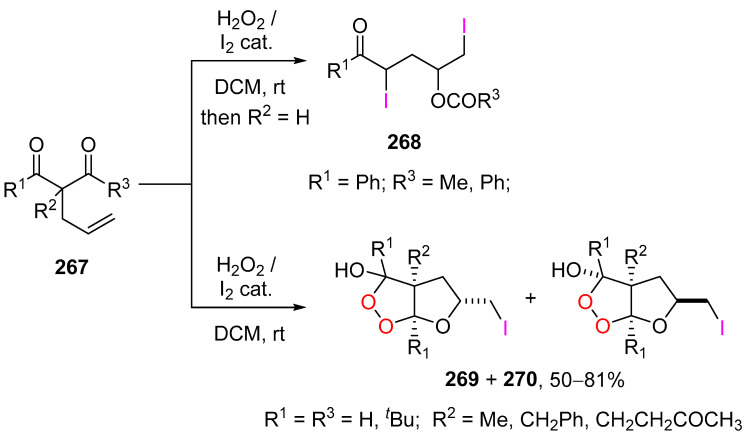
Synthesis of diastereomeric bicyclic peroxides **269** and **270**.

Peroxidation of 2-allyl-1,3-diketones **267** under the action of the I_2_/H_2_O_2_ led to the formation of diastreoisomeric bicyclic peroxides **269** and **270** (Scheme 99) [194]. The reaction was carried out under mild conditions in dichloromethane at 20–25 °C with the use of a five-fold molar excess of H_2_O_2_ and a two-fold excess of I_2_ with respect to the starting diketone. It should be noted that the expected bridged tetraoxanes were not found during the peroxidation of 1,3-diketones **267**. Diastereomeric iodine peroxides **269** and **270** were obtained as a mixture of diastereoisomers with a yield of 50 to 81%. The interaction of ketones **267** containing an aromatic ring adjacent to the carbonyl group with the I_2_/H_2_O_2_ system led to the formation of iodides **268** with a yield of 11–24%, but not to the bicyclic peroxides **269** and **270**.

**Scheme 100:**
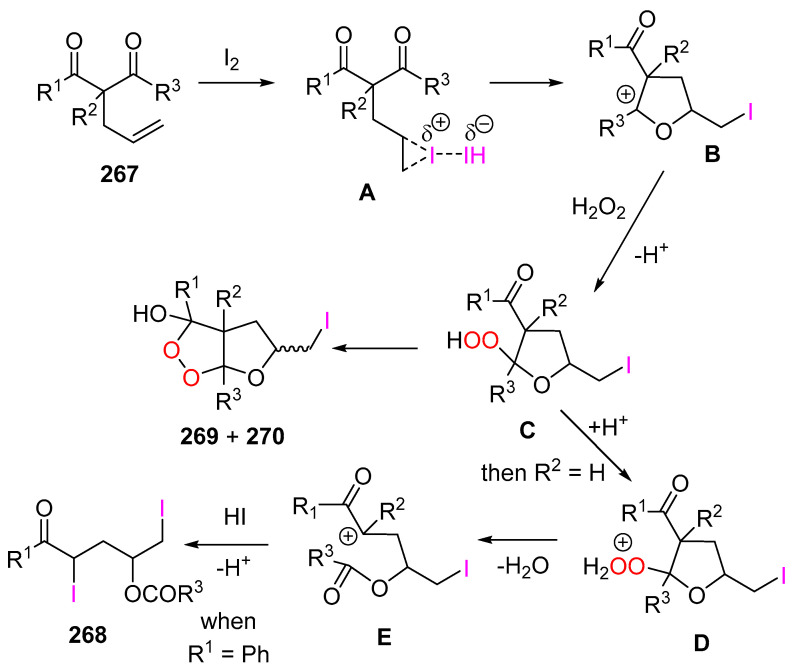
The proposed mechanism for the assembly of bicyclic peroxides **269** and **270**.

The first stage involves the interaction of iodine with a double bond to form the iodonium cation **A**, which undergoes cyclization to the intermediate tetrahydrofuran **B,** stabilized by the anomeric effect [66,67,68] (Scheme 100). Then H_2_O_2_ attacks **B** with the formation of iodoperoxide **C**, which undergoes cyclization with the formation of **269** + **270**. In the case of compounds containing an aryl substituent at the carbonyl group, peroxide **C** is protonated with the formation of **D**, which undergoes Baeyer-Villiger rearrangement to form cation **E**, which is iodinated by HI to form **268**.

**Scheme 101:**
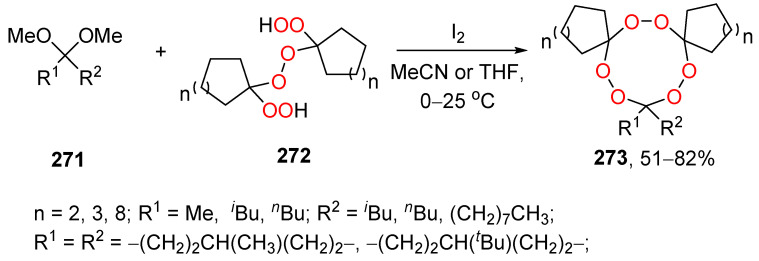
Synthesis of 1,2,4,5,7,8-hexaoxananes **273**.

A method was proposed for the synthesis of 1,2,4,5,7,8-hexaoxananes **273**, based on the I_2_-catalyzed reaction of acetals **271** with 1,1′-dihydroperoxydi(cycloalkyl) peroxides **272** (Scheme 101) [195]. This method allows for the obtaining of cyclic triperoxides in good yields from 51 to 82%.

### 3.3. Synthesis of Peroxides Promoted by TMSOTf and TBDMSOTf

**Scheme 102:**
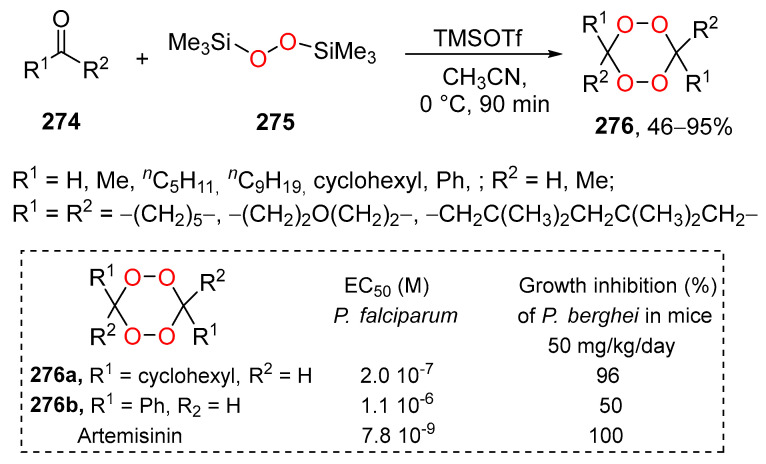
Synthesis and activity of 1,2,4,5-tetraoxanes **276**.

A convenient method has been developed for the synthesis of symmetric 1,2,4,5-tetraoxanes **276** from carbonyl compounds **274** and peroxidizing agent bis(trimethylsilyl) peroxide **275** in the presence of 1 equiv. of TMSOTf. The reaction was carried out at 0 °C in acetonitrile or at −70 °C in CH_2_Cl_2_ (Scheme 102) [196]. The in vitro and in vivo studies demonstrated that these types of cyclic peroxides are active against *P. falciparum* [197].

**Scheme 103:**
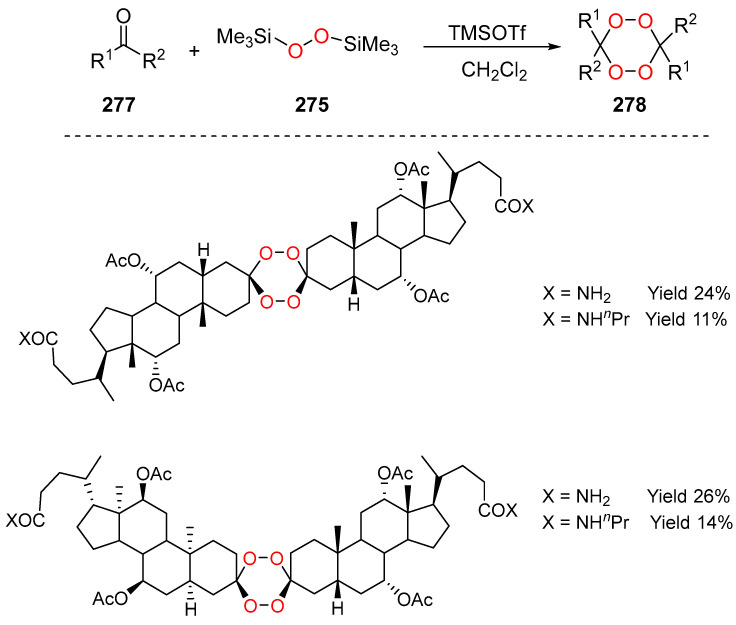
Synthesis of tetraoxanes **278**.

Peroxidation of carbonyl compounds with the use of Me_3_SiOOSiMe_3_/TMSOTf system allows one to obtain steroidal tetraoxanes **278** (Scheme 103) [198]. The reaction was carried out at 0 °C in acetonitrile using a 1.5-fold molar excess of Me_3_SiOOSiMe_3_ and TMSOTf with respect to ketone **277**.

**Scheme 104:**
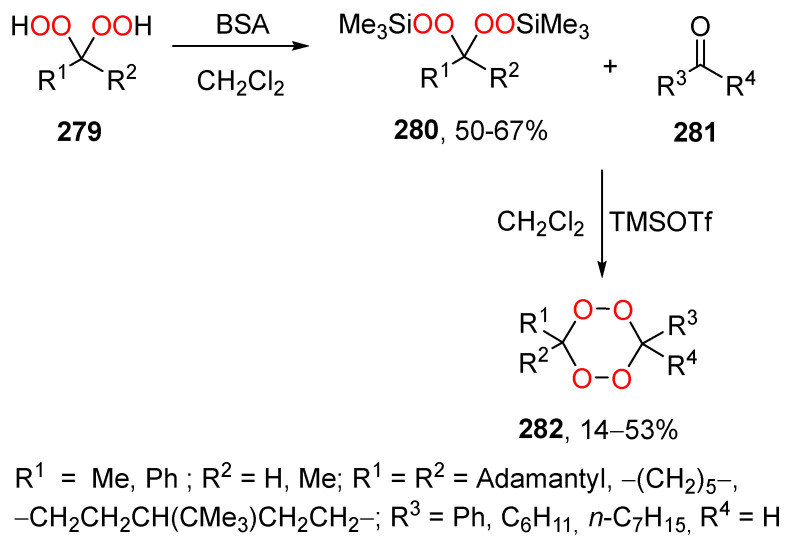
Synthesis of unsymmetrical 1,2,4,5-tetraoxanes **282**.

The synthesis of unsymmetrical 1,2,4,5-tetraoxanes **282** proceeds through the interaction of geminal bis(trimethylsilyl)peroxides **280** with carbonyl compounds **281** in the presence of TMSOTf. 1,2,4,5-Tetraoxanes **282** are formed in yields up to 53% (Scheme 104) [197]. Corresponding bis(trimethylsilyl) peroxides **280** were obtained by the interaction of geminal bishydroperoxides **279** with BSA (N, O-bis (trimethylsilyl) acetamide) in 50–67% yield.

**Scheme 105:**
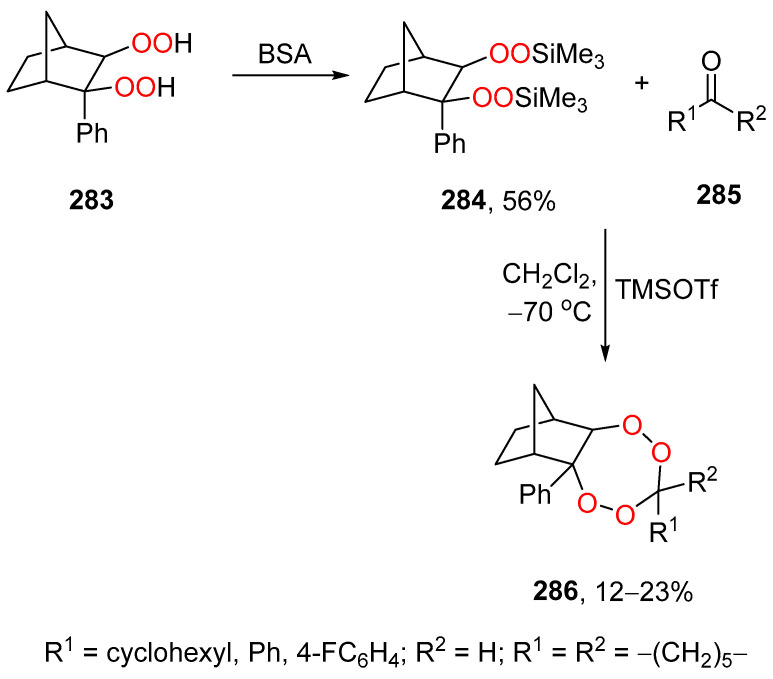
Synthesis of 1,2,4,5-tetraoxepanes **286**.

In addition, TMSOTf is used as a catalyst in the synthesis of 1,2,4,5-tetraoxepanes **286** by the reaction of 1,2-bis (trimethylsilyl) peroxide **284** with carbonyl compounds **285** (Scheme 105) [197,199]. Silyl peroxide **284** was synthesized by reaction of BSA (N, O-bis (trimethylsilyl) acetamide) on 1,2-dihydroperoxide **283** in 56% yield.

**Scheme 106:**
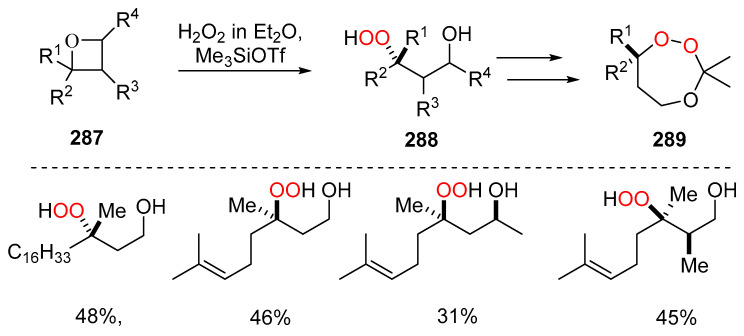
Synthesis of 1,2,4-trioxepanes **289**.

In 2002, Dussault et al. [111,127] demonstrated the oxetane ring opening in **287** with an ether solution of H_2_O_2_, catalyzed by Yb(OTf)_3_, with the formation of 3-hydroxyhydroperoxide **288**, which act as an intermediate in the synthesis of 1,2,4-trioxepanes **289**. However, the use of TMSOTf in some cases led to a better result (Scheme 106).

**Scheme 107:**
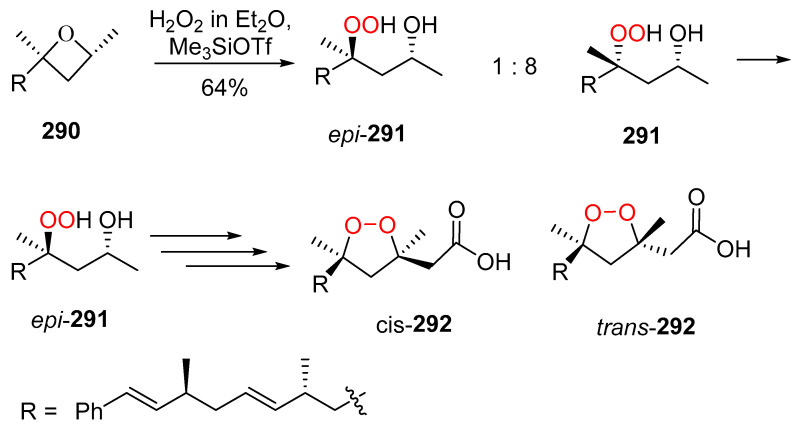
Synthesis 3-hydroxy hydroperoxides **291** and ***epi*-291**.

The developed system TMSOTf/H_2_O_2_ for oxetane ring opening in **290** was successfully used at one of the stages in the total synthesis of plakinic acid A ***cis*-292** and ***tran*s-292**, a natural compound with antitumor activity (Scheme 107) [111]. Opening of the oxetane ring in **290** by TMSOTf resulted in the formation of readily separable 3-hydroxyhydroperoxides **291** and ***epi*-291**. Then, in several steps, cyclic peroxides ***cis***-**292** and ***trans***-**292** were obtained from ***epi*-291**.

**Scheme 108:**
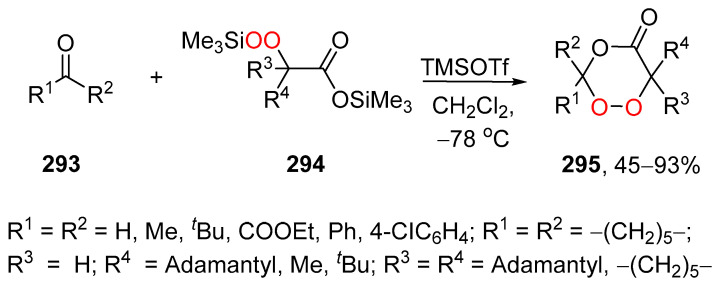
Synthesis of 1,2,4-trioxan-5-ones **292**.

Cyclic peroxolactones (1,2,4-trioxan-5-ones) **295** were obtained by the reaction of carbonyl compounds **293** with silyl peroxides **294** under the action of TfOSiMe_3_. This reaction does not proceed in the absence of TfOSiMe_3_. The synthesis was carried out in methylene chloride at a temperature of −78 °C (Scheme 108) [200,201].

**Scheme 109:**
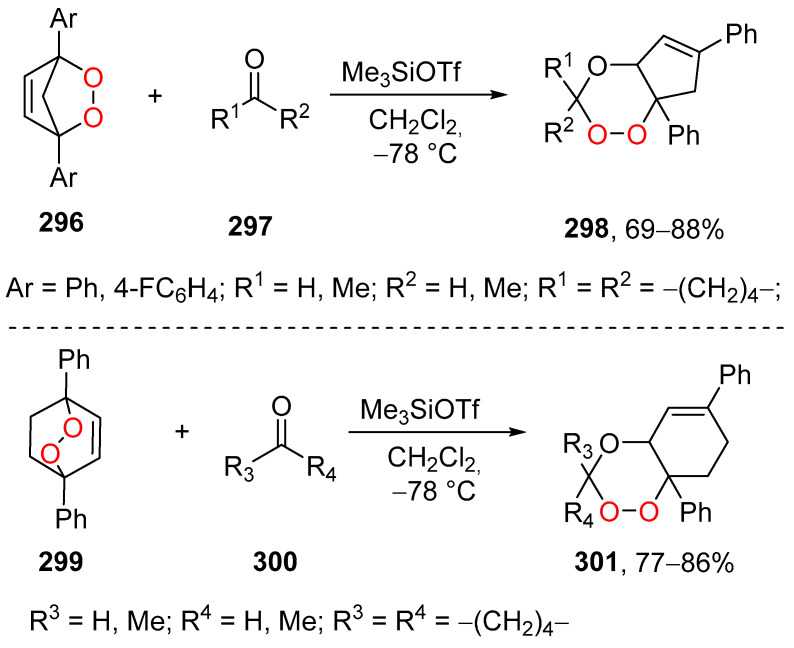
Synthesis of substituted 1,2,4-trioxanes **298** and **301**.

The TMSOTf-catalyzed interaction of endoperoxides **296** and **299** with ketones **297** and **300** proceeds with the formation of above-mentioned substituted 1,2,4-trioxanes **298** and **301** with moderate to good yields (Scheme 109) [202,203,204].

**Scheme 110:**
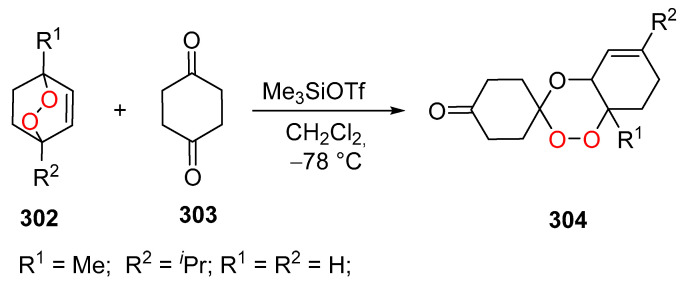
Synthesis of substituted 1,2,4-trioxanes **304**.

1,2,4-Trioxanes **304** were obtained by the reaction of diketone **303** with containing alkyl substituents endoperoxides **302** (Scheme 110) [205]. Unfortunately, the yield of target peroxides did not exceed 10%.

**Scheme 111:**
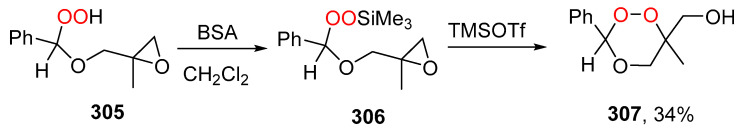
Synthesis of substituted 1,2,4-trioxane **307**.

Trimethylsilyl peroxide **306**, which was obtained by the reaction of BSA (*N*,*O*-bis (trimethylsilyl) acetamide) with hydroperoxide **305**, intramolecularly reacts with the oxirane ring under the action of TMSOTf to form 1,2,4-trioxane **307** in 34% yield (Scheme 111) [206].

**Scheme 112:**
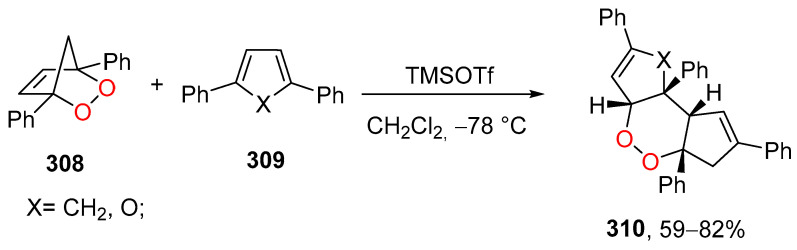
Synthesis of tetrasubstituted 1,2-dioxanes **310**.

The use of TMSOTf in the reaction of endoperoxide **308** with cyclic diene **309** opened access to tetrasubstituted 1,2-dioxanes **310** (Scheme 112) [207].

**Scheme 113:**
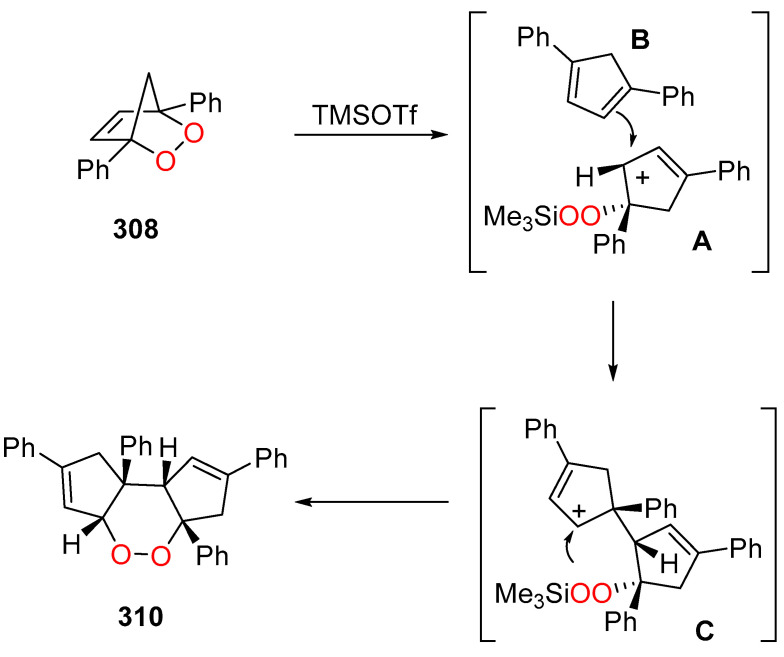
The proposed mechanism for the assembly of tetrasubstituted 1,2-dioxanes **310**.

At the first step, the interaction of endoperoxide **308** with TMSOTf leads to the formation of carbocation **A**. The subsequent attack of 1,4-diphenyl-1,3-cyclodiene **B** on carbocation **A** occurs in a regio- and diastereospecific manner. The intramolecular attack of the peroxysilyl function on the carbocation in **C** leads to product **310** (Scheme 113).

**Scheme 114:**
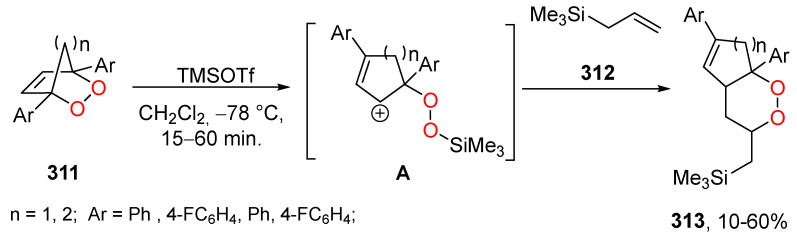
Synthesis of bicyclic 1,2-dioxanes **313**.

The reaction of allyltrimethylsilane **312** with endoperoxides **311** in the presence of catalytic amounts of TMSOTf resulted in bicyclic 1,2-dioxanes **313** with a yield of 10% to 60% (Scheme 114) [208].

**Scheme 115:**
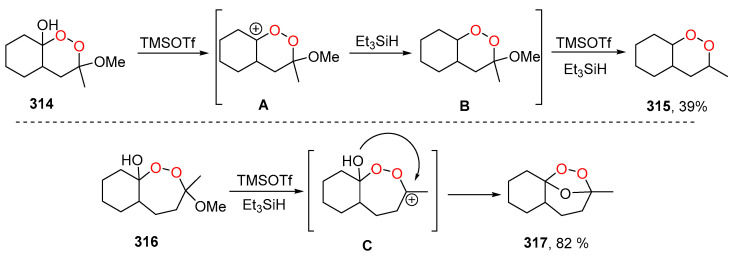
Synthesis of substituted 1,2-dioxane **315** and 1,2,3-trioxolane (ozonide) **317**.

The use of TMSOTf/Et_3_SiH system in the reaction with bicyclic peroxides **314** and **316** led to unusual results. Substituted 1,2-dioxane **314** was transformed into 1,2-dioxane **315**. But in the case of a 7-membered cyclic peroxide **316**, the main product was bicyclic peroxide containing ozonide cycle **317** (Scheme 115) [209].

**Scheme 116:**
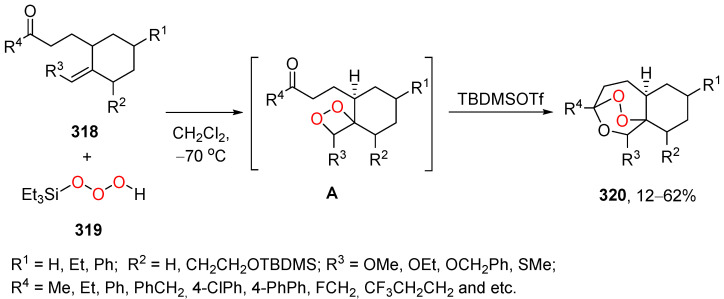
Synthesis of 1,2,4-trioxanes **320**.

It has been shown that triethylsilyl hydrotrioxide **319** (Et_3_SiOOOH), obtained in situ from ozone and triethylsilane, is a mild and effective dioxetane-forming reagent from vinyl ethers and vinyl thioethers on a relatively small (50–100 mg) scale. A number of studies have demonstrated that the interaction of TBDMSOTf with dioxetane **A** leads to its rearrangement into 1,2,4-trioxanes **320**. Such peroxides exhibit in vitro antimalarial activity, which is not inferior to peroxides like Artemisinin (Scheme 116) [210,211,212].

**Scheme 117:**
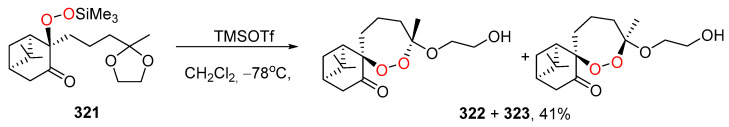
Synthesis of cyclic peroxides **322** and **323**.

In a study [213] on the synthesis of cyclic peroxides **322** and **323** with high antimalarial activity, TMSOTf was used as a catalyst at the stage of peroxide cycle assembly (Scheme 117). Peroxoacetals **322** and **323** were obtained from substrate **321** in 41% yield. The antimalarial activity of peroxides **322** and **323** is comparable to the antimalarial activity of Artemisinin.

## 4. Heteropoly Acids in the Synthesis of Organic Peroxides

In recent years, great interest has been paid to heteropoly acids as catalysts in the synthesis of organic peroxides. Heteropoly acids such as phosphomolybdic (PMA) and phosphotungstic (PTA) acids have a unique ability to form peroxo complexes with hydrogen peroxide and transfer the peroxide function to the substrate [37,214,215,216]. The deposition of heteropoly acids on a support allows them to be reused after regeneration [37,216]. This section covers approaches on the synthesis of bisperoxides, 1,2,4-trioxolanes, 1,2,4,5-tetraoxanes, and tricyclic monoperoxides with the use of heteropoly acids.

**Scheme 118:**
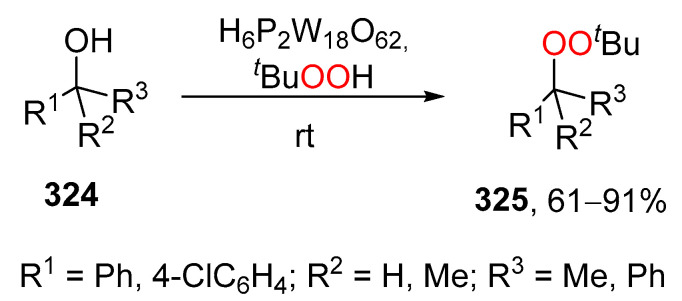
Synthesis of peroxides **325**.

The use of the ^t^BuOOH/H_6_P_2_W_18_O_62_ system allows one to obtain dialkyl peroxides **325** from alcohols **324** in good yield (Scheme 118) [217]. In the case of secondary alcohols, the formation of an ether was observed in the reaction, which led to a decrease in the yield of the target peroxide. No by-product formation was observed in the case of tertiary alcohols.

**Scheme 119:**
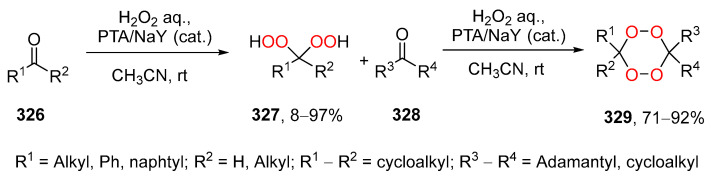
Synthesis of geminal bisperoxides **327** and 1,2,4,5-tetraoxanes **329** on their basis.

Supported phosphotungstic acid (PTA) on zeolite (NaY) allows the synthesis of a wide range of geminal bisperoxides **327** under heterogeneous conditions with a yield of 8 to 97% (Scheme 119) [216]. Such a system (H_2_O_2_, PTA/NaY) is effective for the synthesis of 1,2,4,5-tetraoxanes **329**. Target products **329** were obtained in 71% to 92% yield.

**Scheme 120:**
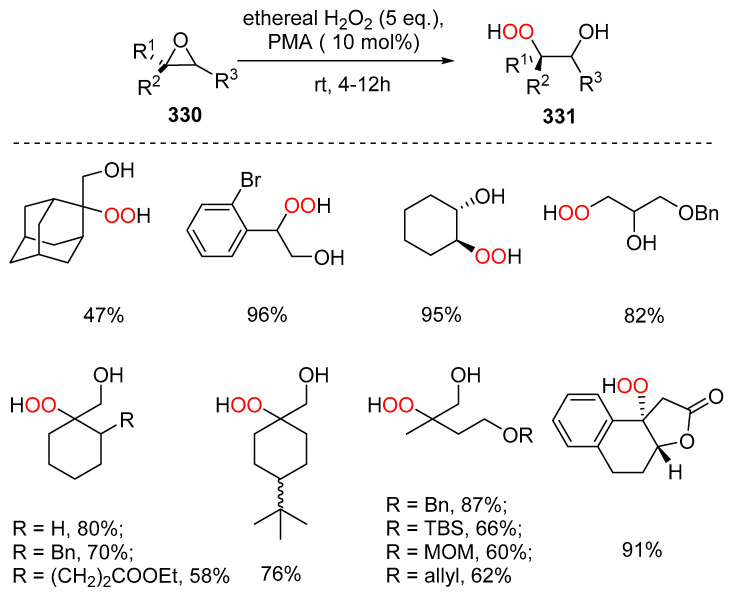
Synthesis of β-hydroperoxy alcohols **331** from epoxides **330**.

In 2009, the group of Wu et. al. reported the application phosphomolybdic acid (PMA) as a catalyst for the ring-opening of epoxides with H_2_O_2_. This method gives the opportunity to obtain β-hydroperoxy alcohols **331** at ambient temperature (Scheme 120) [218]. For all tested substrates the ring-opening of epoxides **330** is highly regioselective to give the hydroperoxyl group at the quaternary carbon.

**Scheme 121:**
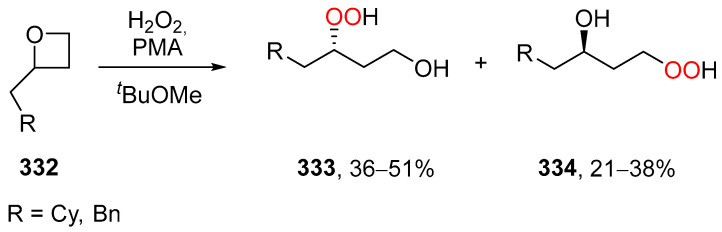
Synthesis of peroxide **333** and **334**.

In 2014, Han et. al. performed the ring opening of oxetane **332** with hydrogen peroxide in the presence of phosphomolybdic acid (PMA). This interaction resulted in a mixture of two peroxides **333** and **334** (Scheme 121) [219].

**Scheme 122:**
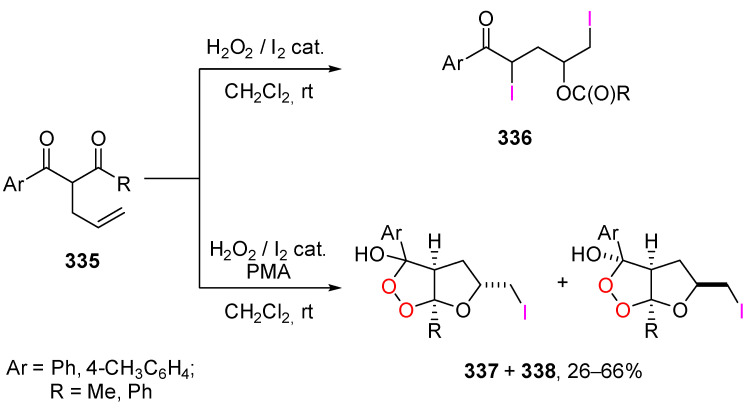
Synthesis of bicyclic peroxides **337** and **338**.

The ability of heteropoly acids to form peroxo complexes and coordinate with the carbonyl group allows the peroxidation of ketones and their derivatives under milder conditions. For example, peroxidation of 1-aryl-2-allylalkane-1,3-diones **335** with I_2_/H_2_O_2_ system proceeds with the formation of iodinated ketoesters **336**. The addition of catalytic amounts of PMA to the I_2_/H_2_O_2_ system facilitates the assembly of bicyclic peroxides **337** and **338** (Scheme 122) [220].

**Scheme 123:**
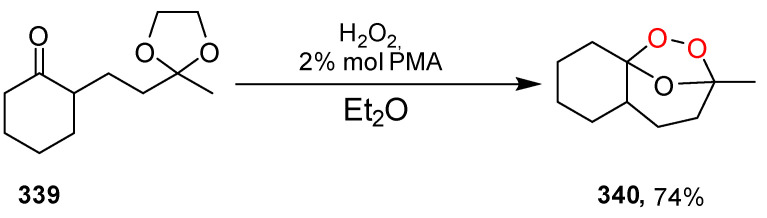
Synthesis of ozonide **340**.

Ozonide **340** was obtained in one step by peroxidation of ketoacetal **339** with a yield of 74%. Phosphoromolybdic acid (PMA) was used as a catalyst in the amount of 0.02 equiv. with respect to **339**. (Scheme 123) [218].

**Scheme 124:**
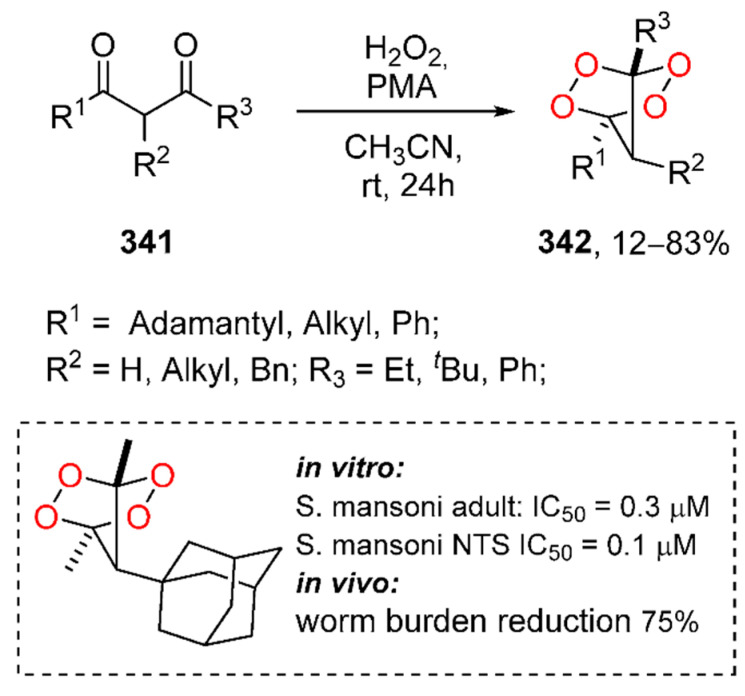
Synthesis of bridged 1,2,4,5-tetraoxanes **342**.

Phosphomolybdic (PMA) and phosphotungstic (PTA) acids efficiently catalyze the peroxidation reaction of β-diketones **341**, including easily oxidized diketones, with the formation of bridged 1,2,4,5-tetraoxanes **342** (Scheme 124) [214]. Peroxides can be obtained in grams. The bridged 1,2,4,5-tetraoxane **342** containing an adamantane substituent in its composition exhibit a high activity (IC_50_: 0.3 μM) in vitro and in vivo (worm burden reduction was 75%) against *S. mansoni* [16].

**Scheme 125:**
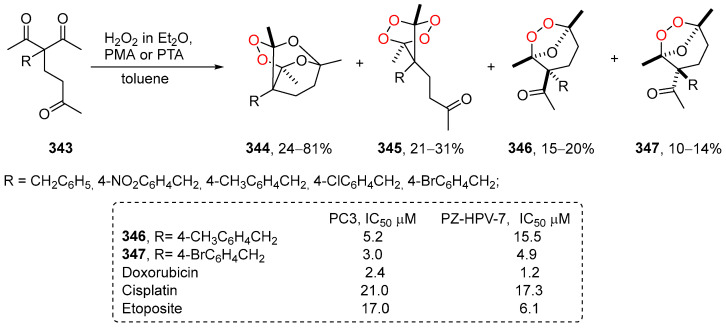
Synthesis and activity of cyclic peroxides **344**–**347**.

The reaction of β,δ’-triketones **343**, containing a benzyl substituent in the α-position, with an ethereal solution of H_2_O_2_, catalyzed by heteropoly acids (PMA, PTA) in a polar aprotic solvent, proceeds along three paths with the formation of three classes of peroxides: tricyclic monoperoxides **344**, bridged tetraoxanes **345** and a pair of stereoisomeric ozonides **346** and **347** (Scheme 125) [215,221]. The reaction is unusual in that bridged tetraoxanes and ozonides with a free carbonyl group were formed. The synthesis of ozonides from ketones and H_2_O_2_ is a unique process in which ozonide is formed with the participation of two carbonyl groups. Bridged ozonides exhibit high in vitro cytotoxicity against androgen dependent prostate cancer cell lines DU145 and PC3. In some cases the anticancer activity of ozonides is higher than that of doxorubicin, cisplatin, and etoposide [222].

**Scheme 126:**
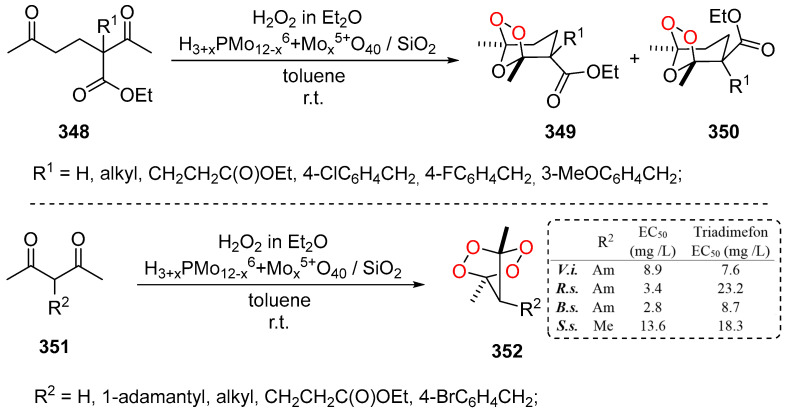
Synthesis of bridging ozonides **349**, **350** and 1,2,4,5-tetraoxanes **352**.

More recently, an efficient catalyst H_3+x_PMo_12-x_^6+^ Mo_x_^5+^O_40_/SiO_2_ was developed for the synthesis of bridged ozonides **349**, **350** and 1,2,4,5-tetraoxanes **352** under heterogeneous conditions (Scheme 126) [37] The synthesis of peroxides under heterogeneous conditions is a rare process and presents a challenge in this area of chemistry, as peroxides tend to decompose on the catalyst surface. The yield of diastereoisomeric bridged ozonides **349**, **350** was up to 90%, and of bridged 1,2,4,5-tetraoxanes **352** wasup to 86%.

## 5. Summary and Outlook

This review summarizes approaches to the synthesis of organic peroxides under the action of Lewis acids and heteropoly acids. The possibility of Lewis acids to coordinate with the oxygen atom of the carbonyl group, as well as to generate a peroxycarbenium ion in the starting compounds, allows for the expansion of the potential of the peroxidation reaction of carbonyl compounds.

The possibility of metal-containing compounds such as PMA, PTA, and MeReO_3_ to form peroxo complexes with hydrogen peroxide makes it possible to transfer the peroxide function to the substrate. This transfer of peroxide groups, mediated by metal complexes, makes it possible to obtain organic peroxides under heterogeneous conditions.

Analysis of the literature allows us to conclude that in the next decade the vector in peroxide chemistry will shift towards the use of the Lewis acid/peroxidizing agent system. This system is promising and its use will open up new horizons in peroxide chemistry for the chemical and medical industries.

## Data Availability

Not applicable.
